# Automated subset identification and characterization pipeline for multidimensional flow and mass cytometry data clustering and visualization

**DOI:** 10.1038/s42003-019-0467-6

**Published:** 2019-06-20

**Authors:** Stephen Meehan, Gleb A. Kolyagin, David Parks, Justin Youngyunpipatkul, Leonore A. Herzenberg, Guenther Walther, Eliver E. B. Ghosn, Darya Y. Orlova

**Affiliations:** 1 Department of Genetics, Stanford University School of Medicine, Stanford, CA 94305 USA; 2 Independent Researcher, Menlo Park, CA 94025 USA; 3 Department of Statistics, Stanford University, Stanford, CA 94305 USA; 4 Departments of Medicine and Pediatrics, Lowance Center for Human Immunology, Emory Vaccine Center, Children’s Healthcare of Atlanta, Emory University, Atlanta, GA 30322 USA

**Keywords:** Statistical methods, Computational platforms and environments

## Abstract

When examining datasets of any dimensionality, researchers frequently aim to identify individual subsets (clusters) of objects within the dataset. The ubiquity of multidimensional data has motivated the replacement of user-guided clustering with fully automated clustering. The fully automated methods are designed to make clustering more accurate, standardized and faster. However, the adoption of these methods is still limited by the lack of intuitive visualization and cluster matching methods that would allow users to readily interpret fully automatically generated clusters. To address these issues, we developed a fully automated subset identification and characterization (SIC) pipeline providing robust cluster matching and data visualization tools for high-dimensional flow/mass cytometry (and other) data. This pipeline automatically (and intuitively) generates two-dimensional representations of high-dimensional datasets that are safe from the curse of dimensionality. This new approach allows more robust and reproducible data analysis,+ facilitating the development of new gold standard practices across laboratories and institutions.

## Abstract (web summary)

Stephen Meehan, Gleb A. Kolyagin et al. present a fully automated subset identification and characterization pipeline for robust cluster matching and data visualization of high-dimensional flow/mass cytometry data. They show that the method can be applied to single- or multi-dimensional data.

## Introduction

The traditional approach to locating clusters (subsets) in high-dimensional (Hi-D) data sets such as those acquired by flow cytometry is to reduce the data set dimensionality, usually by linear and/or nonlinear one-/two-dimensional mapping or projection strategies. This Projection Pursuit approach has proven to be very efficient for analyzing high-dimensional data in a way that avoids a common pitfall, the curse of dimensionality (see refs. ^1,2^; Supplementary Notes 1 and 2, Supplementary Figs 1–3). Indeed, much of what is known about stem cells, blood cells, and diseases, such as leukemia and AIDS, relies on flow-cytometry data analyzed with these manual sequential Projection Pursuit approaches, including the widely used methods offered by FlowJo (www.flowjo.com). Usually, cell subsets identified in such user-guided manners are readily biologically interpretable. However, the resolution of such subsets with manual analysis tools is by no means routine. In fact, since these manual analysis methods ultimately rely on user skills to define subset boundaries, subset identification, and quantitation is still more appropriately recognized as an art rather than a science, and, as such, automating this data analysis process and making it more objective is clearly desirable.

Several groups have recently developed fully automated computational approaches that operate simultaneously in four or more dimensions to identify the subsets (clusters) within a given Hi-D data set^3^. These attempts are well motivated from a functionality point of view. However, there are several issues associated with the fully automated Hi-D clustering approach. First, the reproducibility of clusters automatically generated from simultaneous analysis of multiple dimensions is proving challenging;^4^ as we have shown previously, this irreproducibility is partially caused by the curse of dimensionality (see ref. ^5^; Supplementary Notes 1 and 2, Supplementary Tables 1–3). Second, there is no widely accepted analytical framework to distinguish spurious clusters from more stable entities, and presumably more biologically relevant ones^4^. Finally, there is a lack of tools to readily interpret fully automated clustering outcomes.

To facilitate statistical and biological inference from fully automated (and user-guided) clustering outcomes, we introduced a pipeline of multidimensional cluster matching and display methods. We based our pipeline on the quadratic form distance metric and adaptive binning^6^. We previously demonstrated^6^ that a computationally efficient distance metric such as quadratic form, which takes into account changes in both location and frequency rather than just changes in one or the other, is the most suitable and accurate method for comparing multivariate non-parametric flow/mass cytometry data distributions. In addition, by coupling the quadratic form metric with adaptive binning, we avoid the curse of dimensionality in both cluster matching and data visualization. Together with a clustering algorithm, our methods provide a complete pipeline for cluster (subset) recognition, display, and characterization. The analysis pipeline we describe is readily applicable to any number of dimensions and to any method that enables valid identification of cellular (or other) subsets. Here, we emphasize that it is crucially important to fuse/apply cluster matching and visualization modules to valid methods of subset identification (i.e., those that avoid the curse of dimensionality). We avoid the curse in cluster identification here by coupling cluster matching and data visualization tools with the fully automated Exhaustive Projection Pursuit (EPP) clustering approach available at: www.cytogenie.org; http://cgworkspace.cytogenie.org/GetDown2/demo/bCellMacrophageDiscoveryDemo.pdf; (paper in preparation). Here, we apply these statistically robust clustering and data visualization tools to both simulated and previously published flow/mass cytometry data sets and emphasize that they are readily applicable to similar single- or multidimensional data generated with other technologies.

## Results

### Cluster analysis and data visualization pipeline

**Fig. 1:**
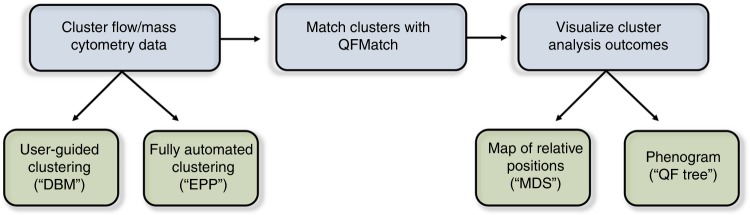
A flowchart displaying the steps of the Subset Identification and Characterization (SIC) pipeline. SIC pipeline does not require that the data be from flow cytometers. However, the data must be numerical (not categorical) and it must be in an fcs file format

In a simplified example (Supplementary Data 1, and 2), we illustrate steps of the cluster analysis and data visualization pipeline (Fig. 1) that we develop.

Here, we used fully automated EPP clustering (www.cytogenie.com) to locate subsets. This clustering method relies on the same principles underlying the previous automated two-dimensional (2D) Exhaustive Projection Pursuit approaches^7^. Briefly, EPP takes the following stepwise approach: Hi-D data are presented as a collection of 2D linear projections; every 2D projection is then characterized by a numerical index that indicates the amount of structure that is present;^7,8^ this index is then used as the basis for a heuristic search to locate the most useful 2D projection; once the projection with the most useful structure has been found, this structure is then segmented and each portion is recursively analyzed until there is no remaining structure detectable.

In general, Projection Pursuit methods are a big step toward solving the problem of Hi-D data analysis because they avoid the curse of dimensionality. However, the approaches advanced thus far have some key limitations. For example, what constitutes structures in data and how to make inferences from such identified structures is neither obvious nor trivial to specify^9^. To overcome these limitations, we are developing (paper in preparation) a fully automated EPP method (its implementation is available at www.cytogenie.org) that uses the smallest misclassification error across a decision boundary between identified clusters (using the DBM approach, see Supplementary Methods^10^) as an index to identify the most profitable 2D projection.

**Fig. 2:**
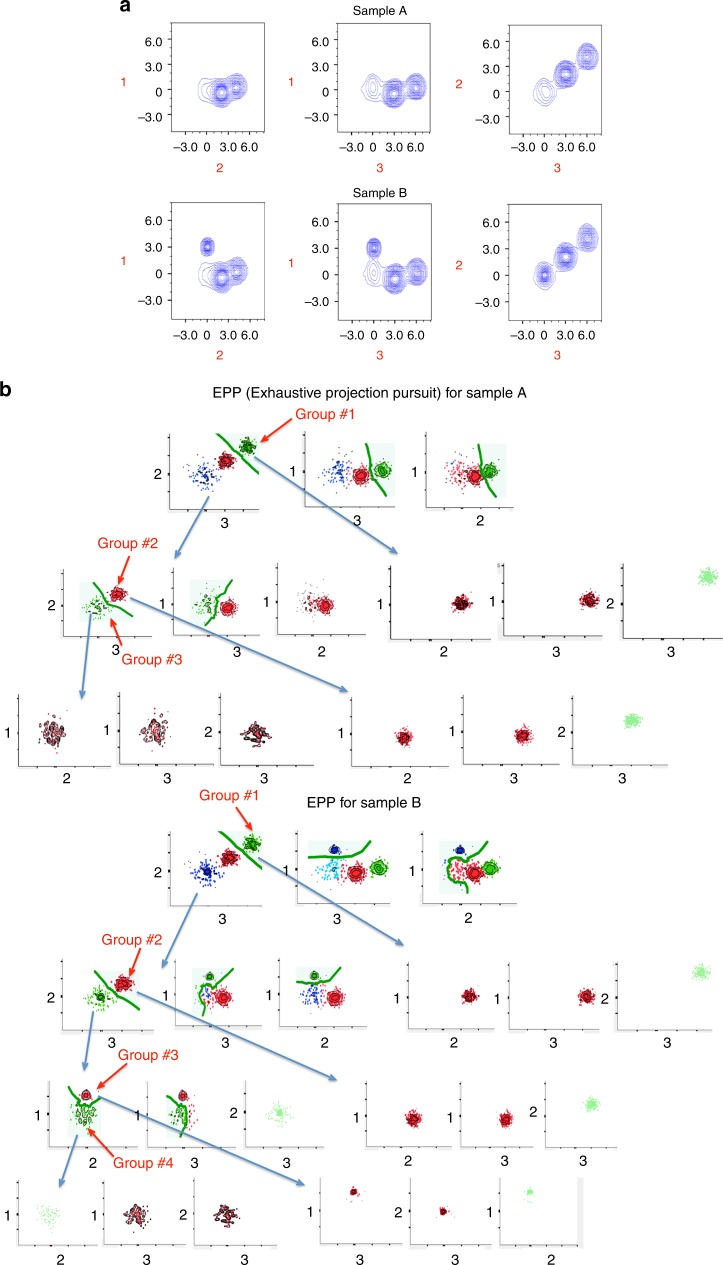
Exhaustive Projection Pursuit (EPP) applied to simulated data set. **a** We simulated two three-dimensional sets of data (Sample A and Sample B). **b** To identify clusters in these data sets, we applied EPP approach that, recursively, (1) projects the data in a collection of 2D linear projections, (2) characterizes every given 2D projection by a numerical index that indicates the smallest misclassification error across a decision boundary (green line) between identified clusters, (3) separates the data across the top-ranked decision boundary to produce two subsets, (4) repeats on both subsets until no splits are found. Subsets that have no further splits (groups# 1–4) are final clusters identified by the EPP approach

The basic strategy underlying the EPP methods is a search for an orthogonal 2D projection, in which the data are cleanly split into subsets. Applied recursively, EPP carries out this strategy, identifying subsets until no further splits are available (Fig. 2). Thus, for a set of measurements, EPP: examines all possible 2D projections using the density-based merging (DBM, see Supplementary Methods)^10^ clustering method and assigns all unclustered data (e.g., outliers) to the nearest cluster; finds all suitable candidate decision boundaries; ranks them by estimated classifier error; separates the data across the top-ranked decision boundary to define two subsets; repeats the above on each of the two subsets until no further splits are found.

**Fig. 3:**
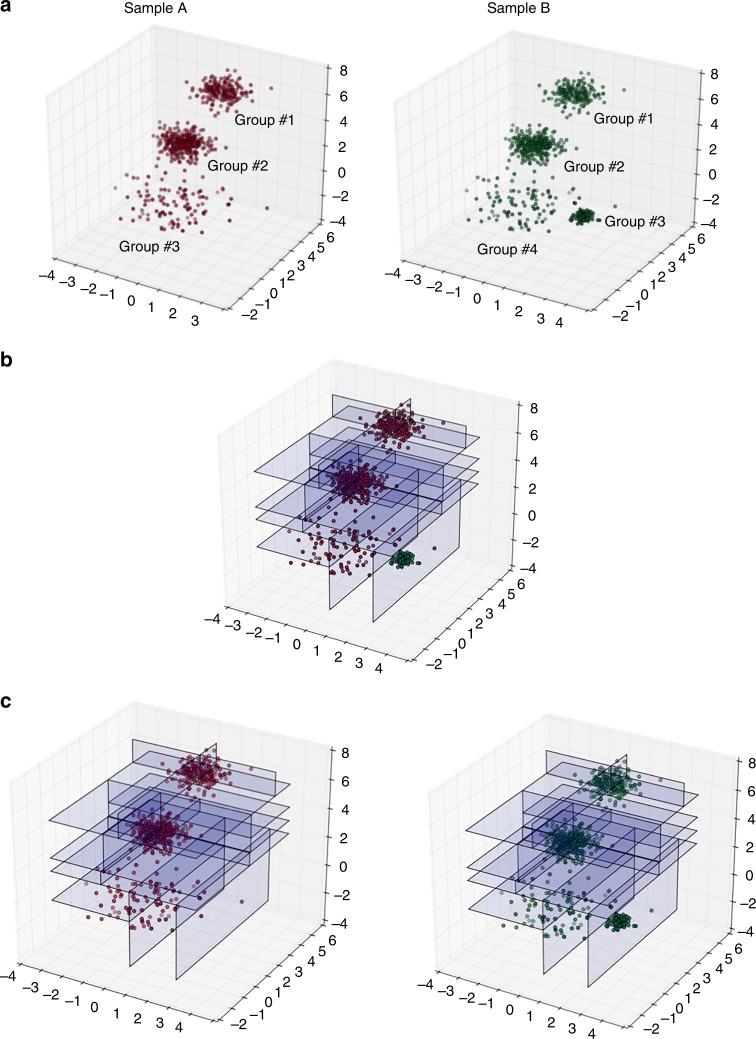
The steps of the QFMatch algorithm as applied in aligning clusters identified by the EPP approach. **a**–**c**. Merge the previously clustered samples (panel **a**, samples were clustered as described in Fig. 2b) and perform adaptive binning (panel **b**) insuring 2ln*N* events per bin, where *N* is the number of events in the smallest sample. Split the samples back while preserving the binning pattern (panel **c**). Calculate quadratic form dissimilarity^6^ between each possible combination of cluster pairs (Groups #1-#4 from Sample A and Sample B), on which medians are located no more than four standard deviations apart in every dimension (Table 1). Pairs with the smallest dissimilarity scores are considered as matched. The remaining clusters in each sample are automatically treated as merging candidates (Table 2). If there is more than one merging candidate then all possible permutations of merging candidates are considered.

**Fig. 4:**
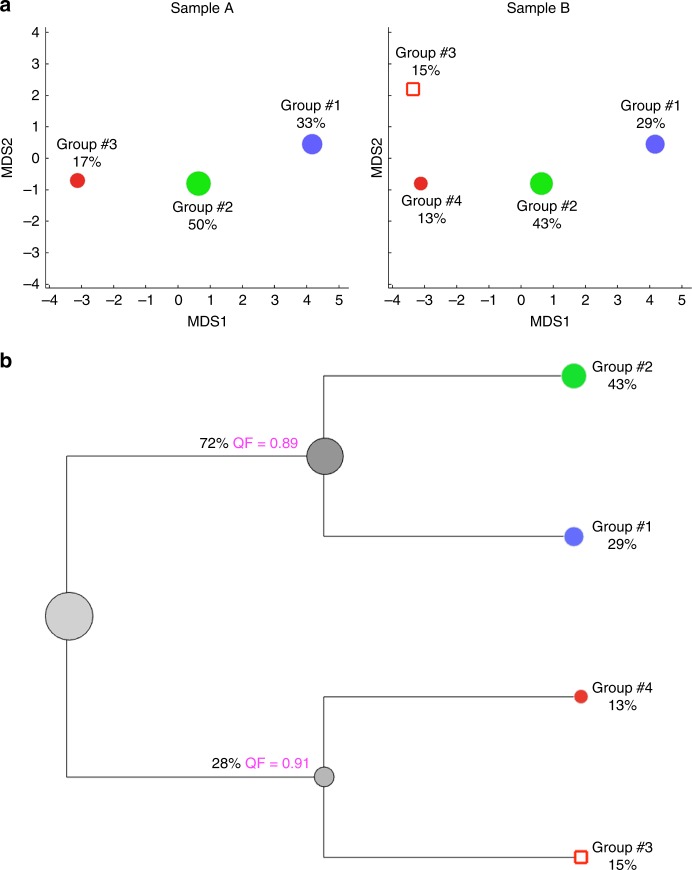
Visualization of cluster analysis outcomes. **a** Display of the EPP clustering outcomes using MDS method. Each circle represents one subset identified by the EPP approach. The size of the circle directly correlates with the relative frequency of the subset in the sample. Subsets that match (identified using QFMatch) between Sample A and Sample B are highlighted with the same color. *X* and *Y* axes are MDS coordinates. We ran MDS on a mixture of Sample A and Sample B to display them in the same *X/Y* scale. Relative location of identified subsets in MDS space corresponds well with the Euclidean distances between subsets’ (groups’) medians presented in Supplementary Table 4. **b** QF (quadratic form)-tree built for Sample B. To build this hierarchical tree from individual clusters, we used the following modification of a multidimensional quadratic form score^6^ as a measure of dissimilarity to progressively merge clusters: quadratic form + c*DM, where *DM* is the Euclidean distance between clusters’ medians and *c* is a scaling factor ensuring that the smallest quadratic form score and the biggest *DM* are numbers of the same order of magnitude. This branching diagram starts by placing clusters with the smallest pairwise dissimilarity scores in the lowest branches of diagram; these pairs of clusters are further progressively merged in the next branching level of the QF-tree and further considered as one cluster; dissimilarity scores are then recalculated for all of the clusters on this branching level and the merging process is repeated. This process is sequentially repeated until all of the clusters identified within the sample are merged together. We named this tree-structure data display as QF-tree

To align (match) subsets identified by EPP (or another clustering algorithm) in two or more comparable samples (e.g., samples A and B in Fig. 2), we used the quadratic form-based cluster-matching algorithm (QFMatch) that we described previously^6,11^. However, here, we extended the previous version of QFMatch by adding an exhaustive cluster merging step. Figure 3a–c illustrates the application of QFMatch to a three-dimensional data set. Matched subsets in samples A and B are highlighted with the same color (Fig. 4a). As we show in the next section, QFMatch can be applied to match subsets identified by different clustering approaches (e.g., user guided versus fully automated, or to compare outcomes between different fully automated algorithms) within one sample.

**Table 1:** Pairwise quadratic form-based dissimilarity scores

Sample B					
**Sample A**	Group ID	1	2	3	4
	1	0.0003 (match)			
	2		0.00004 (match)		
	3		0.86	0.73 (merg.candidate)	0.00016 (match)

Pairs with the smallest dissimilarity scores are marked as “match”. The merging candidate is marked as “merg. candidate”

**Table 2:** The merging process

Sample B				
**Sample A**	Group ID	1	2	3 + 4
	1			
	2			
	3			0.17

If as a result of the merging process the initial dissimilarity score (see Table 1) decreases then the presence of a cluster split is indicated, if not then the unmatched cluster is considered as missing

To visualize clustering outcomes in a composite figure, we developed two data display alternatives that can supplement each other.

We use a multidimensional scaling (MDS) method^12^ that allows placement of each object (cluster) in two-dimensional space such that the overall between-object distances in high-dimensional space are well-preserved. To make the results more visually interpretable, we apply this MDS method to the matrix of distances between median values calculated for each of the identified clusters (Fig. 4a). This reduces the effect of the crowding problem^13^ and, importantly, allows computationally efficient application of MDS.

We also created a tree-structure data display (Fig. 4b) that allows agglomerative arrangement of identified clusters based on their (dis)similarity in the space of measured parameters. This data display method builds the hierarchy from the individual clusters identified within one sample by progressively merging clusters. In order to decide which clusters should be merged, a measure of dissimilarity between sets of observations is required. We used a combination of the multidimensional quadratic form score described in our previous paper^6^ and Euclidean distance between clusters’ medians as a dissimilarity measure to combine identified clusters in a bottom up manner: the branching diagram starts by placing clusters with the smallest pairwise dissimilarity scores in the lowest branches of the diagram; these pairs of clusters are progressively merged in the next branching level of the diagram and then considered as one cluster; dissimilarity scores are then recalculated for all of the clusters on this branching level and the merging process is repeated. This process is sequentially repeated until all of the clusters identified within the sample are merged together. We named this tree-structure data display QF-tree. Building such tree-structure display using quadratic form distance measure + Euclidean distance as a measure of dissimilarity is computationally costlier than using just Euclidean distance. But as we previously demonstrated^6^, distance metrics (such as quadratic form) that take into account changes in both location and frequency rather than just changes in one or the other are the most suitable and accurate methods for comparing multivariate non-parametric flow-cytometry data distributions. Here, we add Euclidean distance to quadratic form distance measure to ensure linear monotonic behavior for this dissimilarity measure (see Fig. 1 in ref. ^6^).

We refer to the above computational pipeline (i.e., clustering for subset identification, QFMatch for high-dimensional cluster matching, and MDS or QF-tree for data display) as the subset identification and characterization (SIC) pipeline. The algorithms constituting this pipeline are available as parts of the AutoGate software, which is freely available for download by not-for-profit users (.edu,.org,.gov) at www.cytogenie.org.

We also provide a source code (python implementation) for the prototypes of high-dimensional cluster matching and data display algorithms (MDS and QF-tree) at https://github.com/dyorlova/QFMatch_MDS_dendrogram. The python implementation provides alternative choices for MDS data display, including the use of median values or adaptive bins that are calculated for each of the identified clusters.

### SIС pipeline identifies the well-known immune cell subsets within the mouse PerС

**Fig. 5:**
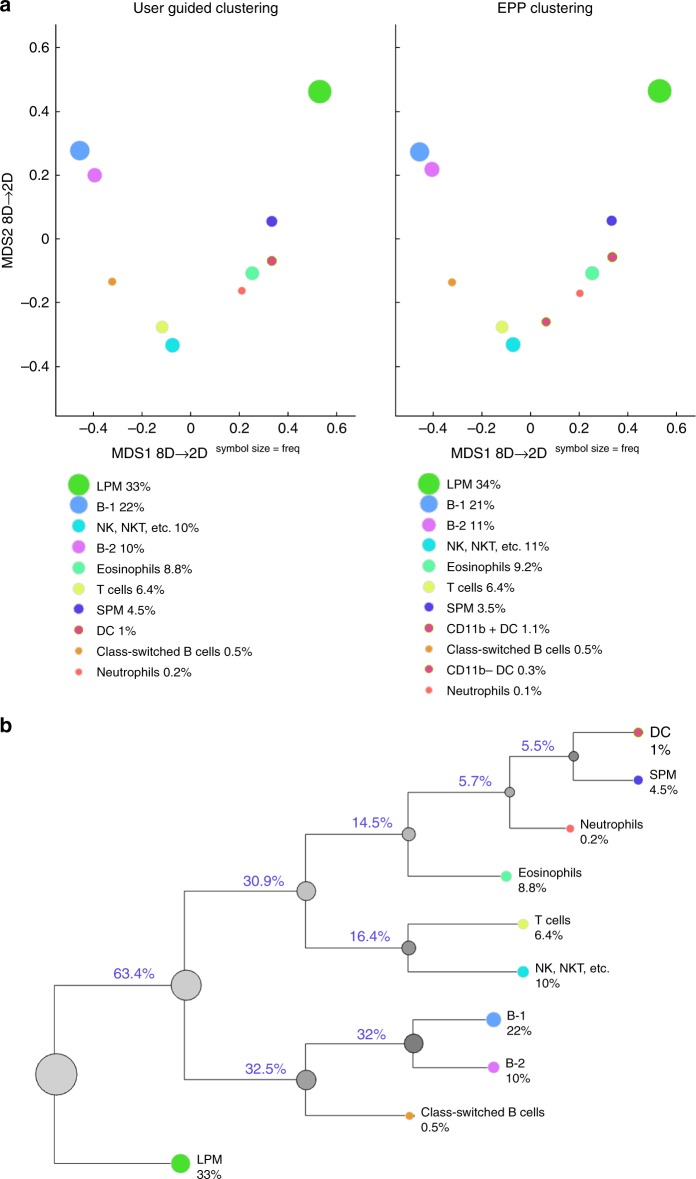
SIC pipeline applied to mouse peritoneal cavity flow-cytometry data. **a** Comparison of conventional (user guided) and EPP (fully automated) clustering outcomes for wild-type (BALB/c) mouse PerC sample as displayed with MDS method (for median fluorescence values comparisons see https://figshare.com/s/9c607084d6bab0d4e1ea, 10.6084/m9.figshare.8115974). EPP clusters were annotated according to multidimensional QFMatch cluster alignment between user-guided and fully automated outcomes in the space of measured parameters (Forward Scatter, CD11b, CD11c, CD19, CD5, F4/80, IgD, and IgM). Each circle corresponds to one identified cell subset, and the size of the circle represents relative cell frequiency. Matched cell subsets are labeled with the same color. **b** QF-tree reflects phenotypic similarity (within the set of measured parameters) between user-guided clustering results for BALB/c mouse PerC sample. The length of edges corresponds to quadratic form score value

To validate the SIC pipeline in a fully automated manner, we applied it to a previously published data set^14^ shown, by manual gating, to contain cells from the myeloid (small and large peritoneal macrophages, and dendritic cells), granuloid (eosinophils and neutrophils), and lymphoid (T, B, NK, and NKT cells) lineages (Supplementary Fig. 4). We show that the standard cell subset measurements (i.e., median fluorescence values and cell frequencies) generated automatically by the SIC pipeline (Fig. 5a; https://figshare.com/s/9c607084d6bab0d4e1ea, 10.6084/m9.figshare.8115974) are in strong agreement with the measurements described by the traditional manual gating method (user-guided clustering) performed by highly skilled investigator^14^.

Such strong agreement in median fluorescence values and cell frequencies between cell populations identified automatically by the SIC pipeline and those identified with user-guided clustering implies their substantial overlap in the space of measured parameters. However, to verify this point, for every cell population identified with the SIC pipeline, we performed backgating analysis to detect the location of the identified cell population within the gating tree built according to a conventional gating strategy. All of the cell populations identified with the EPP approach and matched with those identified by a user-guided approach had a strong overlap (as expected) in their locations on the conventional gating tree (for example, see https://figshare.com/s/9c607084d6bab0d4e1ea, 10.6084/m9.figshare.8115974, where subsets identified with the EPP approach are highlighted in yellow). We also show that the SIC pipeline consistently detects the same immune cell subsets in the peritoneal cavity (PerC) of another wild-type mouse strain (BALB/c) even when a different staining panel is used (see Supplementary Figs 5, 6).

In addition to identifying well-established immune cells subsets, the SIC pipeline was able to identify other cell subsets that were not considered within the established manual gating strategy. For example, using the same set of parameters as in the manual gating strategy, the SIC pipeline identified two subsets of dendritic cells (DC) based on the expression levels of surface CD11b (Supplementary Fig. 7).

Figure 5b shows an agglomerative arrangement (the QF-tree) of the cell subsets (identified by user-guided clustering) according to their (dis)similarity in the space of measured parameters (Forward Scatter, CD11b, CD11c, CD19, CD5, F4/80, IgD, and IgM). In other words, QF-tree organized cells in a hierarchy of related phenotypes. Although QF-tree can reliably reproduce patterns of hematopoiesis from high-dimensional cytometry data, its utility is limited by the choice of markers that are measured in the experiment. For instance, if the tree structure is built with a marker set that is not related to cellular progression, one might not expect to recover the known lineage relationships.

To further test the SIC pipeline performance, we challenged its ability to detect missing lymphocyte populations in the PerC of RAG knockout (RAG−/−) mice. Using QFMatch, we aligned cell subsets identified in the wild-type mice (BALB/c) by the user-guided clustering (Supplementary Fig. 4) with the cell subsets identified in the knockout mice (RAG−/−) by the EPP clustering. The QFMatch algorithm readily matched the non-lymphoid cells present in both BALB/c and RAG−/− samples and correctly detected the lack of T and B lymphocytes in the RAG−/− sample (Supplementary Fig. 8).

### SIС pipeline identifies various subsets of human peripheral B lymphocytes

Using two samples of human peripheral blood stained with the same panel of surface markers (Supplementary Fig. 9), we explored the SIC pipeline’s ability to consistently detect the various lymphoid, myeloid, and granuloid subsets. We used the manual gating strategy shown in Supplementary Fig. 9 to identify T cells, neutrophils, monocytes, naive B cells, memory B cells, class-switched B cells, and transitional B cells. We further used the QFMatch algorithm to align these subsets with the subsets identified by fully automated EPP clustering.

**Fig. 6:**
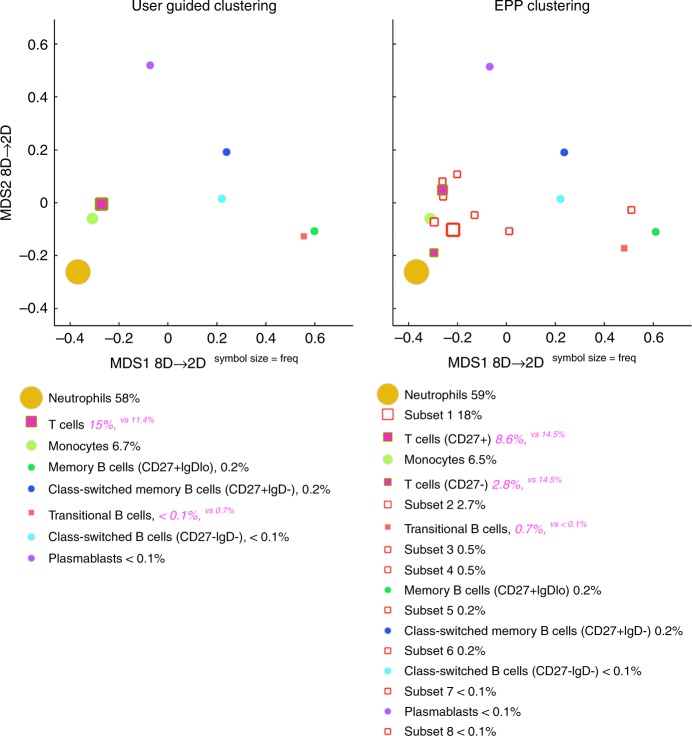
SIC pipeline applied to human peripheral blood flow-cytometry data. The results of multidimensional QFMatch alignment between user-guided and fully automated clustering outcomes for one of the samples (~200k live singlets). The following sets of measured parameters were used for user-guided clustering, EPP clustering, cluster matching and data visualization of pregated live singlets: Side Scatter, Dump (CD3, CD14, CD16), CD19, CD20, CD38, CD27, IgM, IgD. Unmatched cell subsets are indicated as red squares. These unmatched subsets are cell populations that were not identified by the user in the manual gating strategy. User’s gating strategy was not exhaustive, i.e., it did not aim to identify all of the subsets present in the sample, and was limited to identification of the cell populations listed on the left panel. In contrast, EPP is an exhaustive subset identification technique, i.e., all of the subsets present in the sample were identified. These unmatched subsets are cell subsets that were not identified by the user in the conventional gating strategy, but they can now be readily explored looking at: **a** the expression level in each channel via pathfinder tool (see Supplementary Fig. 7); **b** the gating strategy that EPP built (see Supplementary Fig. 11b); **c** backgating with the highlighter tool (see Supplementary Fig. 7). This toolkit (**a**–**c**) was designed to interpret the fully automated clustering outcomes and assign cell subset names to identified clusters. Also, this toolkit can help reveal the presence of a false cluster created by the EPP approach. Essentially, this is a strategy that can be applied to identify and characterize new cell subsets using the SIC pipeline

The QFMatch algorithm successfully aligned the immune cell subsets that were identified in a user-guided manner with those that were identified by a fully automated EPP. QFMatch also reported additional subsets that were not identified manually, but were readily discriminated by EPP clustering (marked as red squares on MDS display, Fig. 6). The SIC pipeline consistently detected all the cell subsets that were identified by the manual gating strategy (Fig. 6; Supplementary Fig. 10).

### SIС pipeline readily identifies human HSCs in the bone marrow

**Fig. 7:**
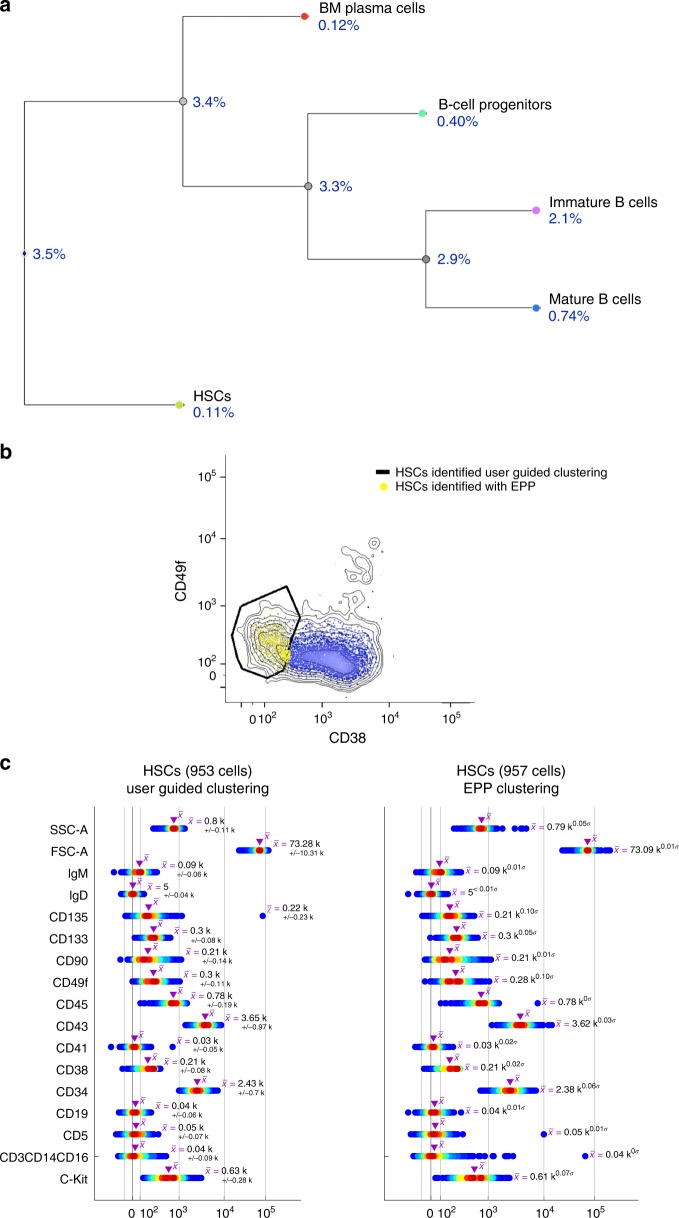
SIC pipeline applied to human bone marrow flow-cytometry data. **a** QF-tree reflects phenotypic similarity (within the set of parameters used in the conventional gating strategy) between the user-guided clustering results obtained with convential gating strategy (Supplementary Fig. 11a). **b** Fully automated clustering (EPP) readily explores the full staining panel (sixteen colors) and finds the most optimal gating strategy (Supplementary Fig. 11b) leading to clear separatation of HSCs subset. **c** HSCs subsets identified automatically by the SIC pipeline are in strong agreement (median fluorescence values and cell frequencies) with those identified with user-guided clustering

Traditionally, human hematopoietic stem cells (HSCs) are characterized by lineage negative, CD34 + , CD38−, CD90 + cells^15^. However, subsequent studies have shown that this population is still heterogeneous and is, at best, enriched for HSCs. Other markers, including CD49f, have been suggested^16^. But, as shown in ref. ^16^ and here in Fig. 7 and Supplementary Fig. 11a, CD49f alone is not sufficient to provide a clear separation of CD49f + HSCs from other cells, limiting the ability to isolate and study highly purified human HSCs.

Here, we applied an unbiased SIC pipeline in which all parameters are used to determine the best markers (and gating strategies) that clearly separate phenotypically distinct subsets. The SIC pipeline readily identified human HSCs in the bone marrow (Fig. 7), defined as lineage negative, CD34 + , CD38−, CD90 + , CD49f + . As shown in Supplementary Fig. 11b, the pipeline used the CD135 marker to better separate HSCs from other progenitor cells.

### SIC pipeline applied to CyTOF clinical samples

We tested the SIC pipeline on the publicly available CyTOF (mass cytometry) data set collected from patients with acute myeloid leukemia (AML) in a pathophysiology study^17^ to illustrate one of the possible applications of the SIC pipeline in clinical/biomedical studies: detection and quantification of a difference in subset representation between healthy controls and AML patients’ samples.

**Fig. 8:**
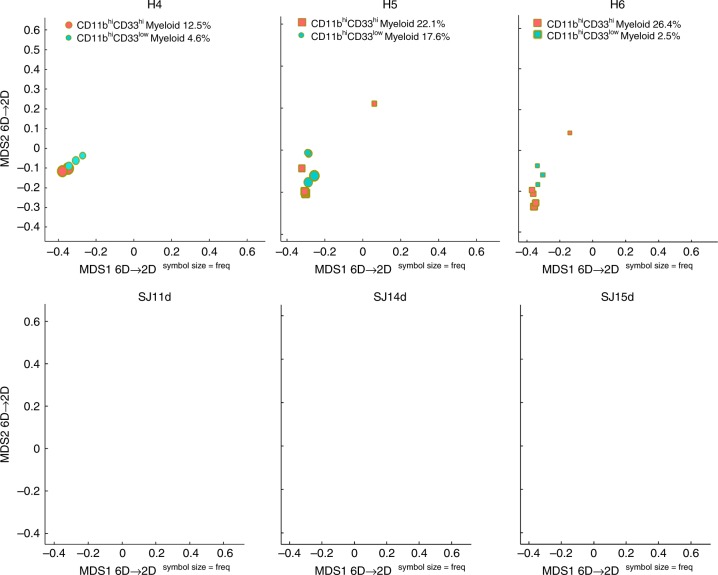
SIC pipeline applied to human bone marrow mass-cytometry data. SIC pipeline reveals the absence of CD11b^hi^ myeloid cells in the AML patients (SJ11d, SJ14d, and SJ15d). However, these patients still maintain their CD33^+^ and CD11b^low^ myeloid cells (see ref. ^22^ for further details)

We randomly selected three healthy controls (H) and three AML patients (SJ) samples from the original study^17^ and compared the representation of CD11b^hi^CD33^low^ and CD11b^hi^CD33^hi^ myeloid cell subsets between these samples. Supplementary Figure 12 (Healthy control) shows the gating strategy used to identify the two myeloid cell subsets in sample H4 with user-guided clustering. We then ran fully automated EPP clustering on all six samples (H4, H5, H6, SJ11d, SJ14d, and SJ15d) individually and matched the clustering outcomes for these samples with the user-guided clustering outcomes for sample H4. Figure 8 shows the difference (relative frequency) in representation of CD11b^hi^CD33^low^ and CD11b^hi^CD33^hi^ myeloid cell subsets between healthy controls and AML patients. To validate the SIC pipeline performance, we further performed user-guided clustering according to the gating strategy shown in Supplementary Fig. 12 (AML patient) to verify whether CD11b^hi^ myeloid cells are absent in the AML patients. SIC pipeline results shown in Fig. 8 correspond very well with the results obtained with the user-guided clustering (see Supplementary Fig. 13).

## Discussion

Modern multidimensional flow- and mass-cytometry data undoubtedly require automation of its analysis. However, despite the decent amount of efforts that have been recently made to automate subset identification and characterization in flow-/mass-cytometry data, these automated methods were not widely adopted among biologists/clinicians. The majority of flow/mass cytometry users still prefer manual gating (e.g., using FlowJo) to automated clustering. One of the main reasons (apart from the vulnerability to the curse of dimensionality) for the lack of adoption of automated methods is their inability to display and align clustering outcomes in a way that allows automatic extraction of meaningful and readily applicable biologically/biomedically information. It is indeed a nontrivial challenge to present the Hi-D clustering results in a way that is easy to understand and interpret and then further align these clustering outcomes between samples or between different clustering algorithms.

Several methods creating two-dimensional visualization of high (or low)-dimensional clustering outomes (e.g., viSNE/tSNE^18^, SPADE^19^) have been developed to aid biologists interpreting Hi-D cytometry data. These methods can provide rich information about the high-dimensional relationship in the data. However, they have some major drawbacks. Both viSNE/tSNE^18^ and SPADE^19^ are prone to suffering from the curse of dimensionality (see Supplementary Notes 1 and 2, Supplementary Figs 14–16) and most importantly they require user-defined input parameters that seriously affect clustering and visualization outcomes. In addition, as noted by Yang et al.^20^ “…[viSNE and SPADE] are not intuitive to biologists who are accustomed to the two-dimensional nested gating representations.”

Aiming to make clustering outcomes more intuitive to biologists, Yang et al.^20^ have recently developed the C2G data visualization method. This method is able to generate a gating hierarchy that captures the target populations (identified by any clustering method) and present the hierarchy in nested two-dimensional gating sequences that resemble the conventional manual gating analysis. Presenting Hi-D data in a two-dimensional nested tree structure may indeed help biologists who are accustomed to the two-dimensional nested gating representations. However, conventional (domain knowledge-driven) gating strategies do not always follow the best separation path that underlies the gating strategy principle applied by the C2G method^20^. Thus, a gating strategy that is built according to the best separation principle may not be readily interpretable to biologists. Moreover, it is not clear how to readily align the trees that followed different gating strategies (e.g., trees that were built from two different samples).

To facilitate statistical and biological inference from automated clustering outomes, we present a pipeline of fully automated, statistically robust cluster matching and data visualization tools applicable to high- (or low-) dimensional data generated with flow-/mass-cytometry and other technologies. The analysis pipeline that we developed here consists of three modules: (1) cluster matching with QFMatch, and (2–3) two-dimensional display of cluster identification and cluster matching results with MDS and/or QF-tree. We designed the cluster matching and data visualization algorithms in a way that automatically produces intuitive representations of Hi-D single-cell data while avoiding the curse of dimensionality. Also, these methods do not require user-defined tuning parameters and their utility is mainly limited by the choice/availability of markers unsed in a particular staining panel (e.g., QF-tree will not retrieve the known lineage relationships if the chosen/available stainset panel does not include key markers related to the cellular progression).

However, while the SIC pipeline provides statistically robust cluster analysis tools, it still has a drawback that is common for all analysis pipelines that rely on fully automated clustering: the inability to automatically assign cell type to identified cluster. This issue can potentially be resolved by parsing the literature for known cell phenotypes and using this data to automatically name clusters that were identified in our fully automated pipeline. But there is no currently established gold standard database that could be used for these purposes. OMIP database (https://onlinelibrary.wiley.com/doi/pdf/10.1002/cyto.a.20916) is an initial effort toward creating a collection of peer-reviewed and readily accessible optimized panels to identify specific cell types, but it is so far limited to just a few panels/cell types.

To mitigate this issue here, we supplement the SIC pipeline with a toolkit (discussed in detail in the legend to Fig. 6) allowing to readily make sense from fully automated clustering outcomes and to assign cell subset names to identified clusters. We successfully applied the SIC pipeline to both user-guided and fully automated clustering outcomes using both flow and mass-cytometry data sets from mouse and human/clinical samples. We implemented this pipeline in the AutoGate (www.cytonegie.org) software package, which supports a graphical user interface, and the Python source code is provided at https://github.com/dyorlova/QFMatch_MDS_dendrogram.

## Methods

### Experiment overview

We use DBM^10^ or EPP (www.cytonegie.org) to identify cell subsets in simulated and flow-/mass-cytometry data, QFMatch^6^ to align subsets between relevant samples (same staining panels), and MDS^12^ or QF-tree to visualize user-guided and fully automated clustering outcomes within the biological/biomedical data sets described below.

### Flow/mass sample description

The mouse peritoneal cavity (Fig. 5) and human bone marrow (Fig. 8) data sets were generated in previously published studies (see refs. ^14,17^ for complete materials and methods).

The human peripheral blood data set (Fig. 6) was generated using a combination of 16 monoclonal antibodies (Hi-D 18-parameter flow cytometry panel): B220-PE (BD Biosciences, catalog # 553090), CD5-PE-Cy5 (BD Biosciences, catalog # 555354), CD10-PerCP-Cy5.5 (BD Biosciences, catalog # 563508), CD19-BV785 (BioLegend, catalog # 302240), CD20-BV650 (BioLegend, catalog # 302336), CD23-APCCy7 (BioLegend, catalog # 338520), CD27-BV421 (BioLegend, catalog # 356418), CD38-APC (BD Biosciences, catalog # 555462), CD43-AF700 (BD Biosciences, catalog # 551457), CD95-BV605 (BioLegend, catalog # 305628), CD132-BV711 (BD Biosciences, catalog # 563129), CD3/CD14/CD16 (Dump)-BV570 (BioLegend, catalog # 300436/BioLegend, catalog # 301832/BioLegend, catalog # 302036), CD45-AF488 (BioLegend, catalog # 304017), IgD-PECy7 (BD Biosciences, catalog # 561314), IgM-PECF594 (BD Biosciences, catalog # 562539), and Aqua Amine (viability) (BioLegend, catalog # 423102). After informed consent, 10 mL of peripheral blood was drawn in evacuated tubes containing EDTA (K2) (Vacutainer, BD Biosciences). The blood samples from healthy adult volunteers were collected, de-identified, and kindly provided by the Clinical and Translational Discovery Core (Biorepository) at Emory University and Children’s Healthcare of Atlanta (http://www.pedsresearch.org/research/cores/biorepository). These studies were exempt from the IRB review because they do not meet the definition of clinical investigation or research with human subjects. The data were collected for about 0,5 × 10^−6^ cells. No data were excluded.

The human bone marrow data set (Fig. 7) was generated using a combination of 16 monoclonal antibodies (Hi-D 18-parameter flow-cytometry panel): CD133-VioBright (Miltenyi Biotec, catalog # 130–113–673), CD49f-BV421 (BD Biosciences, catalog # 747725), IgM-BV570 (BioLegend, catalog # 314517), CD135-biotin (Qdot 605-SA) (BioLegend, catalog # 313312), CD45-BV650 (BioLegend, catalog # 304044), CD5-BV711 (BD Biosciences, catalog # 563170), CD19-BV785 (BioLegend, catalog # 302240), CD3/CD14/CD16 (Dump)-AF647 (BioLegend, catalog # 300416/ BioLegend, catalog # 325612/ BioLegend, catalog # 302020), IgD-AF700 (BioLegend, catalog # 348230), CD43-APC-H7 (BD Biosciences, catalog # 655407), CD34-PE (BD Biosciences, catalog # 345802), C-Kit-PECF594 (BD Biosciences, catalog # 562407), CD41a-PE-Cy5 (BD Biosciences, catalog # 559768), CD38-PerCP-Cy5.5 (BD Biosciences, catalog # 551400), CD90-PE-Cy7 (BioLegend, catalog # 328124), Zombie Aqua (BioLegend, catalog # 423102). The optimal antibody concentration was determined by in-house serial titration using human total peripheral blood. Fresh bone marrow mononuclear cells from adult healthy donors were obtained commercially from AllCells, LLC (Quincy, MA, Cat # ABM024). Data was collected for about 0.5–1.0 × 10^−6^ cells.

### Instrument details

Information about instruments used to collect human and mouse samples can be found in refs. ^14,17^. Human peripheral blood and bone marrow cells were analyzed on the BD LSRII instrument (5-laser, 18-color) at the Emory’s Pediatrics/Winship Flow Cytometry Core.

### Data analysis details

The proposed workflow for analyzing all four data sets used in this paper consists of three steps: Step 1. Transform the compensated data (FlowJo v.10, fluorescence flow-cytometry data only) with the Logicle transformation^21^, and cluster the transformed data with DBM (user guided) [10, see Supplementary Methods] or EPP (fully automated) clustering methods. The data transformation and clustering utilities are available in AutoGate (www.cytonegie.org). The data were pregated for live singlets before EPP clustering run. See figures for gating sequences. The flow-/mass-cytometry data processing methods used here do not require user input for parameters such as the number of clusters, the number of grid bins, etc. Step 2. Use QFMatch to align cell populations between samples or between different clustering outcomes for the same sample. The QFMatch (quadratic form-based cluster-matching algorithm) is integrated into AutoGate (www.cytogenie.org). Step 3. Use MDS and/or QF-tree to display clustering and cluster-matching outcomes. Both visualization tools are integrated into AutoGate (www.cytogenie.org).

QFMatch, MDS, and QF-tree require only one user input configuration parameter, that is the set of markers (and/or light scatter signals) selected to match and display clustering outcomes. Notably, QFMatch, MDS, and QF-tree work independently of how the populations (clusters) were pre-defined. For example, the clusters could be defined by using domain knowledge-driven manual gating, a sequential automated clustering approach, or a simultaneous clustering approach. For details regarding computational performance of QFMatch, MDS and QF-tree methods refer to Supplementary Note 3.

### Reporting summary

Further information on research design is available in the Nature Research Reporting Summary linked to this article.

## Supplementary information

Supplementary Information

Description of Additional Supplementary Files

Supplementary Data 1

Supplementary Data 2

Reporting Summary

## Data Availability

The data sets generated during and/or analyzed during the current study (Figs. 5–8) are available in the FlowRepository and Cytobank: https://flowrepository.org/id/RvFrin9QJBrBl7euVYafJg8MBtow5TSn0Cbf6ibJFTQbutUCP8VbTKi70DJD7TJg, https://flowrepository.org/id/RvFrmp0uY05bFrRfQW6XgcLV360pTCjz5ieEKzaHHGsTDoWEWpBspy21QVrQhFxz, https://flowrepository.org/id/RvFr85dLvWpwdnNDGBkj5qCB8skivxce0qGYsDNtsb52uflvb6C21xDjujsOXnY8, https://www.cytobank.org/nolanlab/reports/Levine2015.html

## Code availability

The analysis pipeline described here is implemented in the AutoGate (www.cytonegie.org) software package, which supports graphical user interface; Python source code is available at: https://github.com/dyorlova/QFMatch_MDS_dendrogram.
