# Supplementary material for: Automated subset identification and characterization pipeline for multidimensional flow and mass cytometry data clustering and visualization
Source: Commun Biol. 2019 Jun 20;2:229. doi: 10.1038/s42003-019-0467-6 (PMC6586874; doi:10.1038/s42003-019-0467-6)
Supplement: Supplementary file 1 — Supplementary Information [file 42003_2019_467_MOESM1_ESM.pdf]

Supplementary Figures

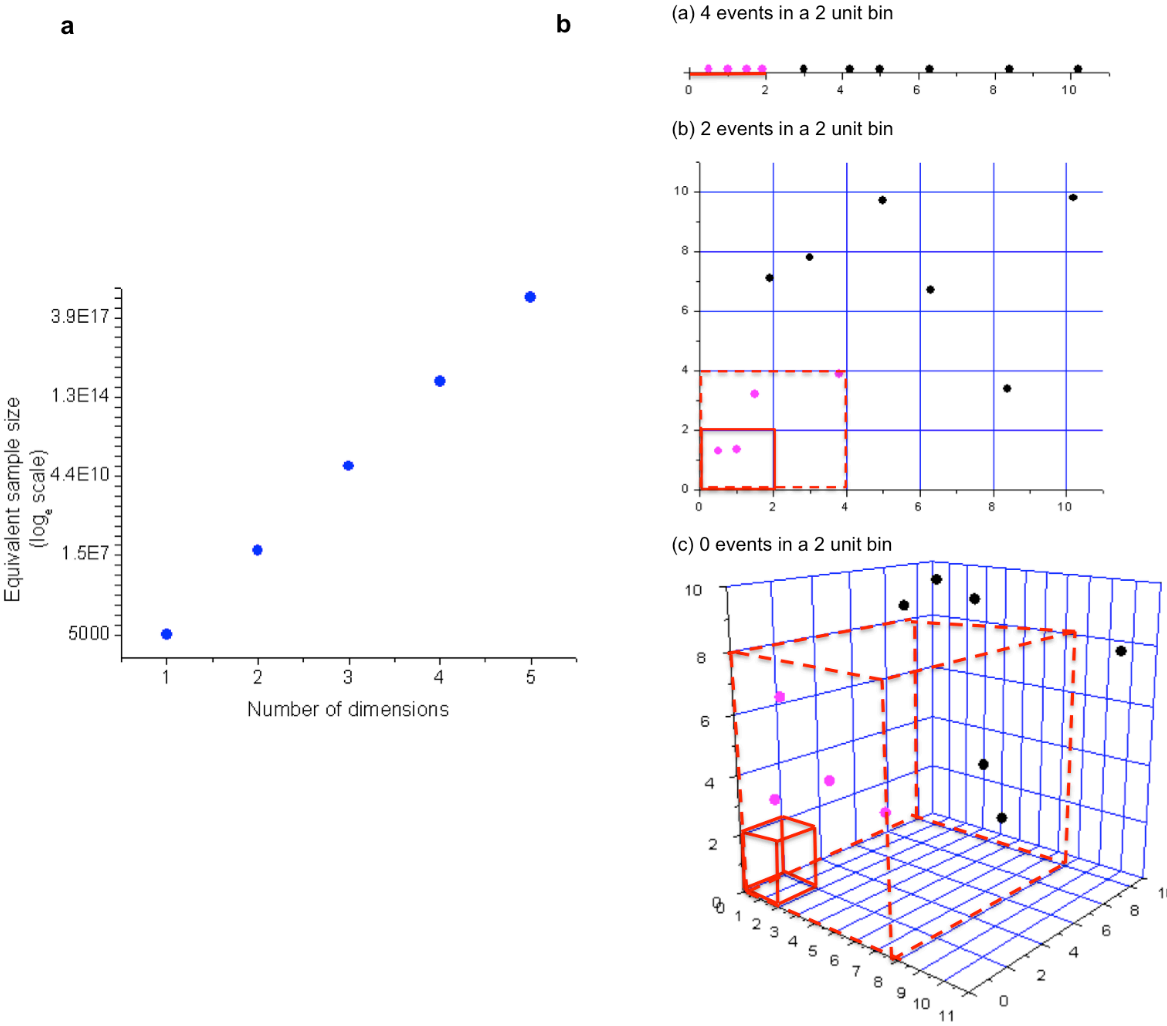

Supplementary Figure 1. The equivalent sample size increases *exponentially* with dimension since a cube with a given side length will contain a progressively shrinking fraction of the observations. Panel a. D.W. Scott [6] shows that in order to reach a given accuracy for typical Hi-D methods, the sample size has to increase exponentially with dimension. For example, since monocytes constitute ~0.5% of spleen cells, based on the expressed level of one biomarker (i.e., one dimension),

locating a population of 25 splenic monocytes from wild-type B6-IgHa mice (dataset is available at https://flowrepository.org/id/FR-FCM-ZZJF) requires data for at least 5000 spleen cells. If monocytes carry three significant biomarkers, then simultaneous Hi-D clustering becomes prohibitive, since the required sample size would be about 1.3 x 10^{11}, i.e., the necessary sample size increases exponentially with dimension such that, for three dimensions, it would be 5000^3, while the total number of cells in the spleen of a wild-type B6-IgHa mouse is about 7.0 x 10^7 . **Panel b.** To locate and operate on data clusters within a multidimensional dataset, data are typically distributed into a multidimensional structure that has *p* dimensions (e.g., fluorescence colors), each of which is represented on a separate axis that has a total of *t* equally spaced ticks. This forms a multidimensional grid in which the whole space is divided in t^p multidimensional boxes. To locate clusters (subsets) in this grid, we estimate the density for each of these multidimensional "boxes" by counting the measured events that fall into it, and then apply a threshold rule to determine which boxes have a high enough density to be scored as "positive".

Unfortunately, for data such as that collected with flow cytometry, this type of density estimation in high dimensional space is well known not to work for accurately identifying clusters. Most boxes simply have too few (or zero, like on panel (c)) observations to enable statistically valid density estimates. For example, a 20-parameter dataset in which there are 100 ticks per dimension (parameter) will have 100^{20} boxes in which the collected data are distributed. Since the number of feasible measurements for flow cytometry data is usually no greater than 1-10 million, even the boxes with the highest

density will typically contain only a few observations, leading to large statistical uncertainties. Therefore it will not be possible to locate clusters in a reliable way.

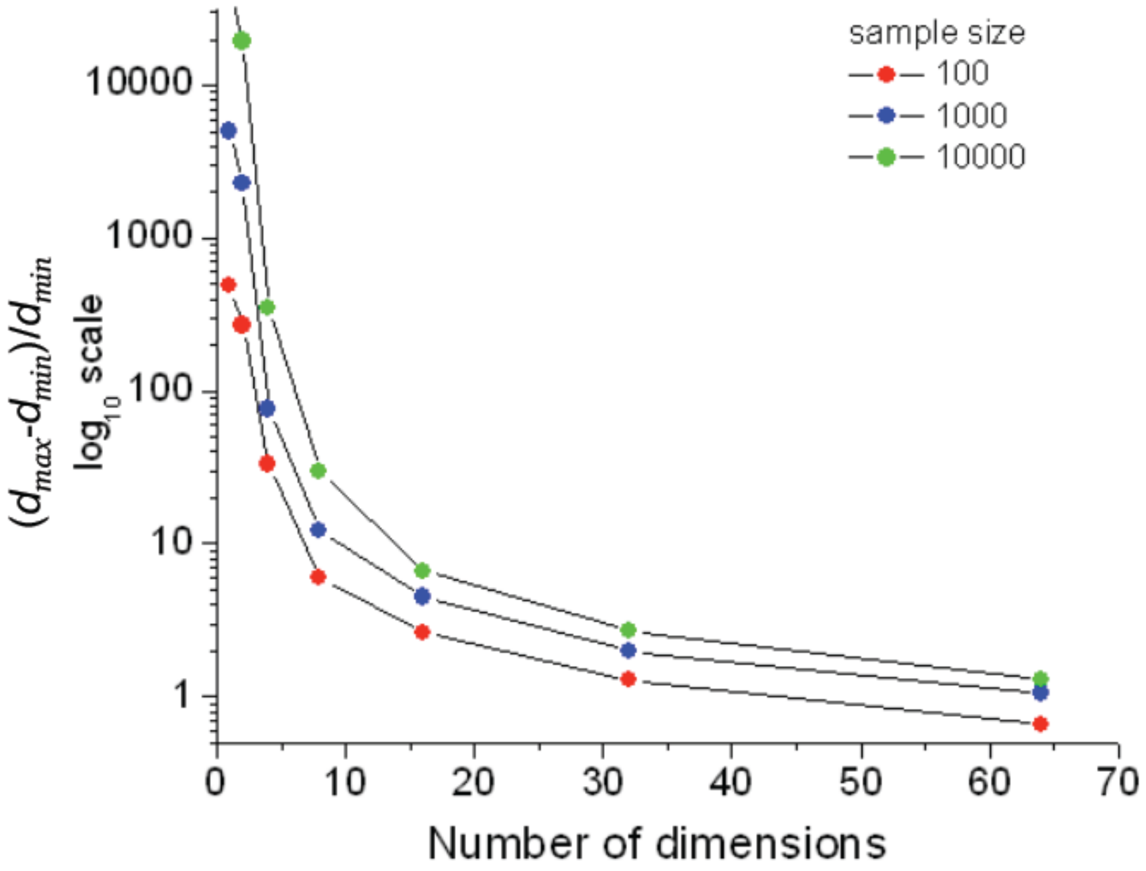

Supplementary Figure 2. Distance measures start losing their effectiveness to measure dissimilarity in high dimensional spaces. We simulated sets of data that are uniformly distributed on a unit cube in varying number of dimensions. For each set we computed the maximum (d_{max}) and minimum (d_{min}) of all pairwise distances between the points. While the ratio of (d_{max} -d_{min})/d_{min} is large in low dimensions, it quickly drops towards 1 as the number of dimensions increases. Therefore, in the latter situation there is essentially no difference between d_{min} and d_{max}, and thus standard procedures which use these distances to detect pattern in the data become useless. This phenomenon

persists even when the sample size is increased by orders of magnitude, e.g., from 100 to 10^4.

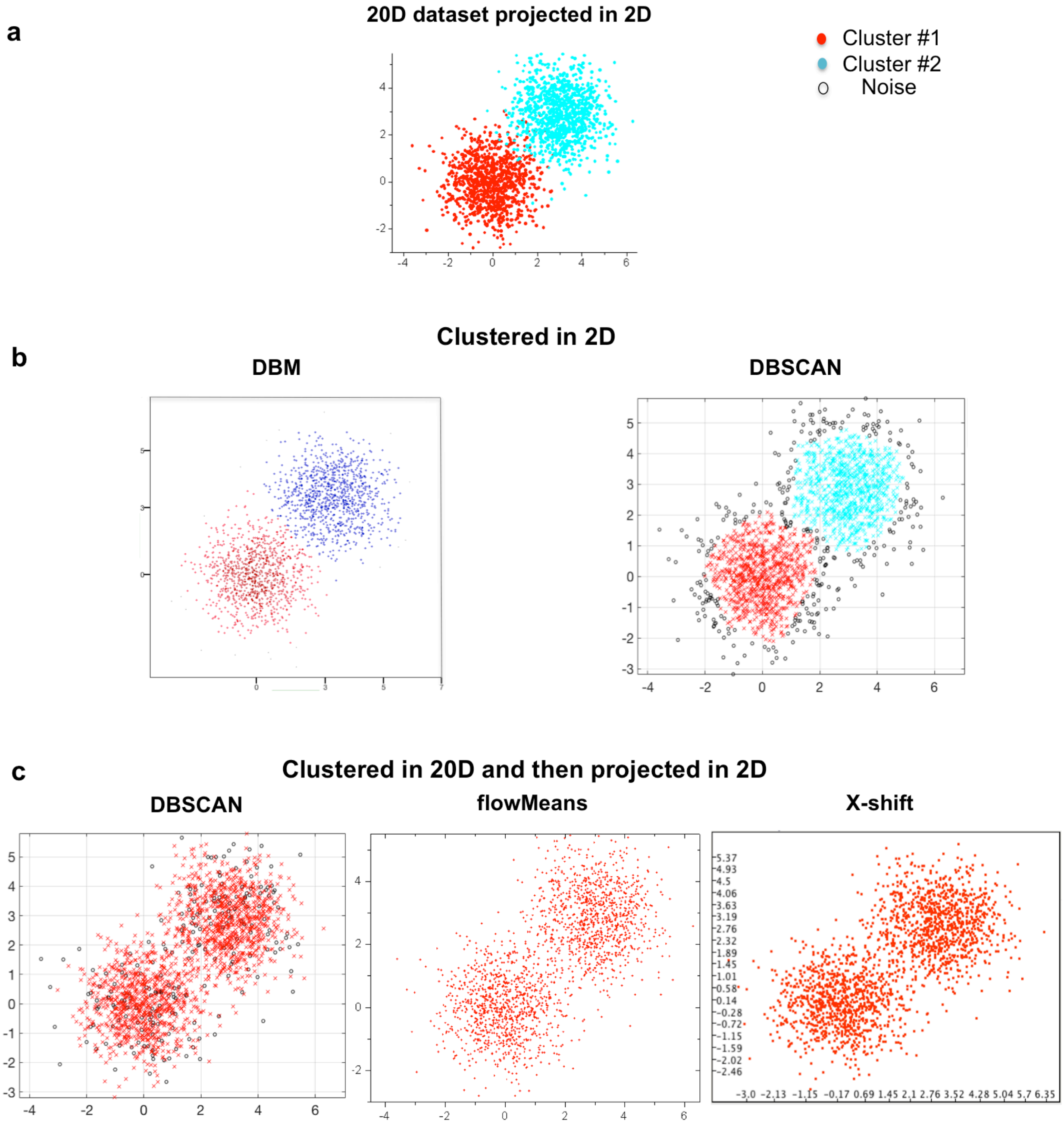

Supplementary Figure 3. High dimensional clustering methods fail to detect two well-separated Gaussian distributions due to the curse of dimensionality. Panel a. A simulated mixture of two 20D Gaussian distributions, one of which is centered at the origin and the other is shifted by 3 units in two of the 20 dimensions. The plot shows the projection into these two dimensions with the two populations being color-coded. The

two populations can be well separated in this projection. **Panel b.** The results of applying DBM [25] and DBSCAN [7] in this 2D projection are coded by color. Both methods do a good job in separating the two populations. The choice of optimal DBSCAN clustering parameters (epsilon and MinPts) was done according to Rahmah et al [26] and [27]. **Panel c.** DBSCAN [7], flowMeans [8,9], and X-shift [10,11] were run on the 20D data. The output was color-coded and then projected into the two dimensions used in panels A and B. The results of these clustering methods show no connection to the actual population structure in the data.

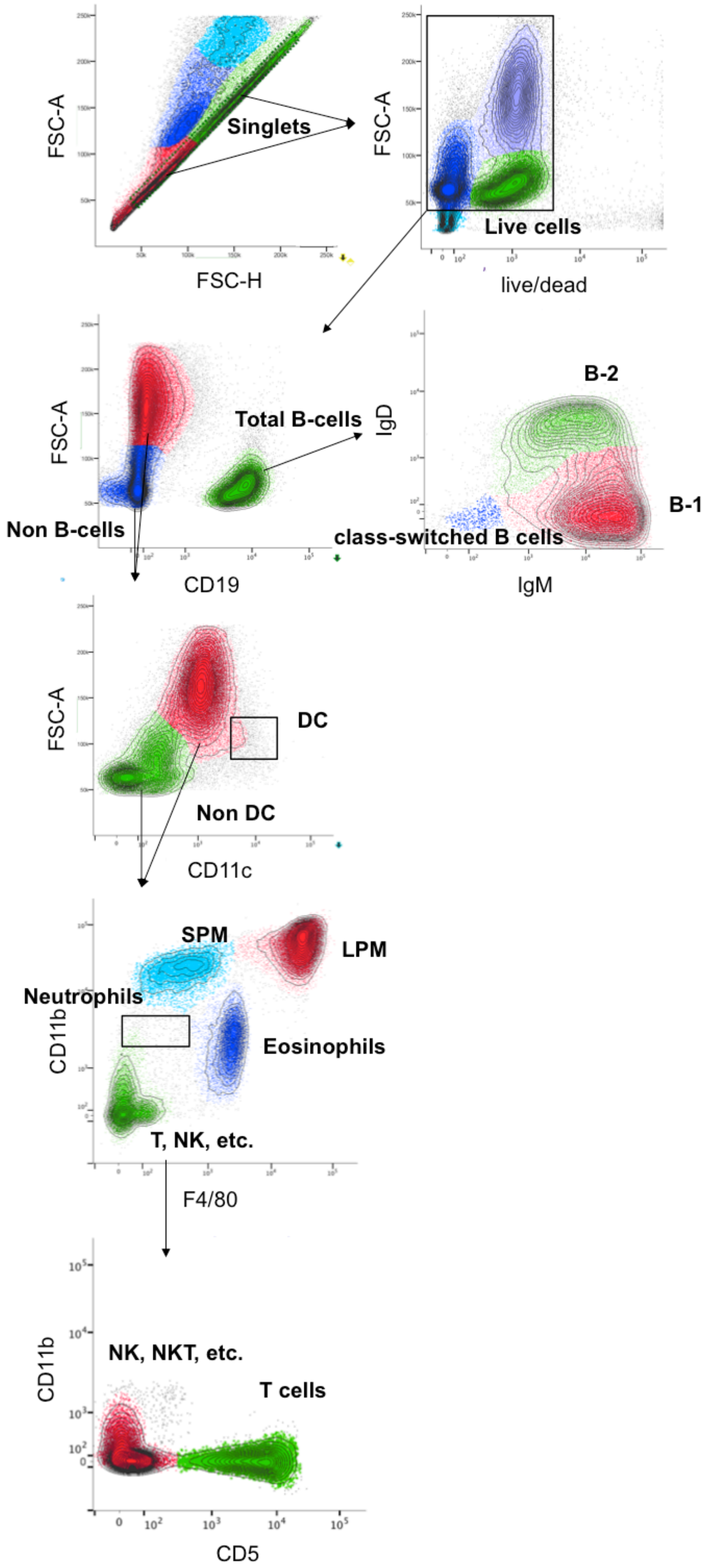
FSC-A
FSC-H
Singlets
FSC-A
live/dead
Live cells
FSC-A
CD19
Total B-cells
Non B-cells
IgD
IgM
B-2
B-1
class-switched B cells
FSC-A
CD11c
DC
Non DC
CD11b
F4/80
SPM
LPM
Neutrophils
Eosinophils
T, NK, etc.
CD11b
CD5
NK, NKT, etc.
T cells

Supplementary Figure 4. Conventional gating strategy used to identify well-known subsets of mouse peritoneal cavity (PerC) cells with user guided clustering. Data shown here were generated in a previously published study [28]. Data show total peritoneal cells from adult (~8 week-old) naïve unmanipulated BALB/c mice. Cells suspensions were stained with a panel of 11 antibodies (11-colors, 13-parameter) to identify the various subsets of tissue-resident immune cells. The goal of this study was to identify and characterize the macrophage heterogeneity in the mouse peritoneal cavity, which are extensively used in biomedical research as a source of primary macrophages. Data were collected for 0.2 to 1 x 10^6 cells. The data is representative of > 10 replicates.

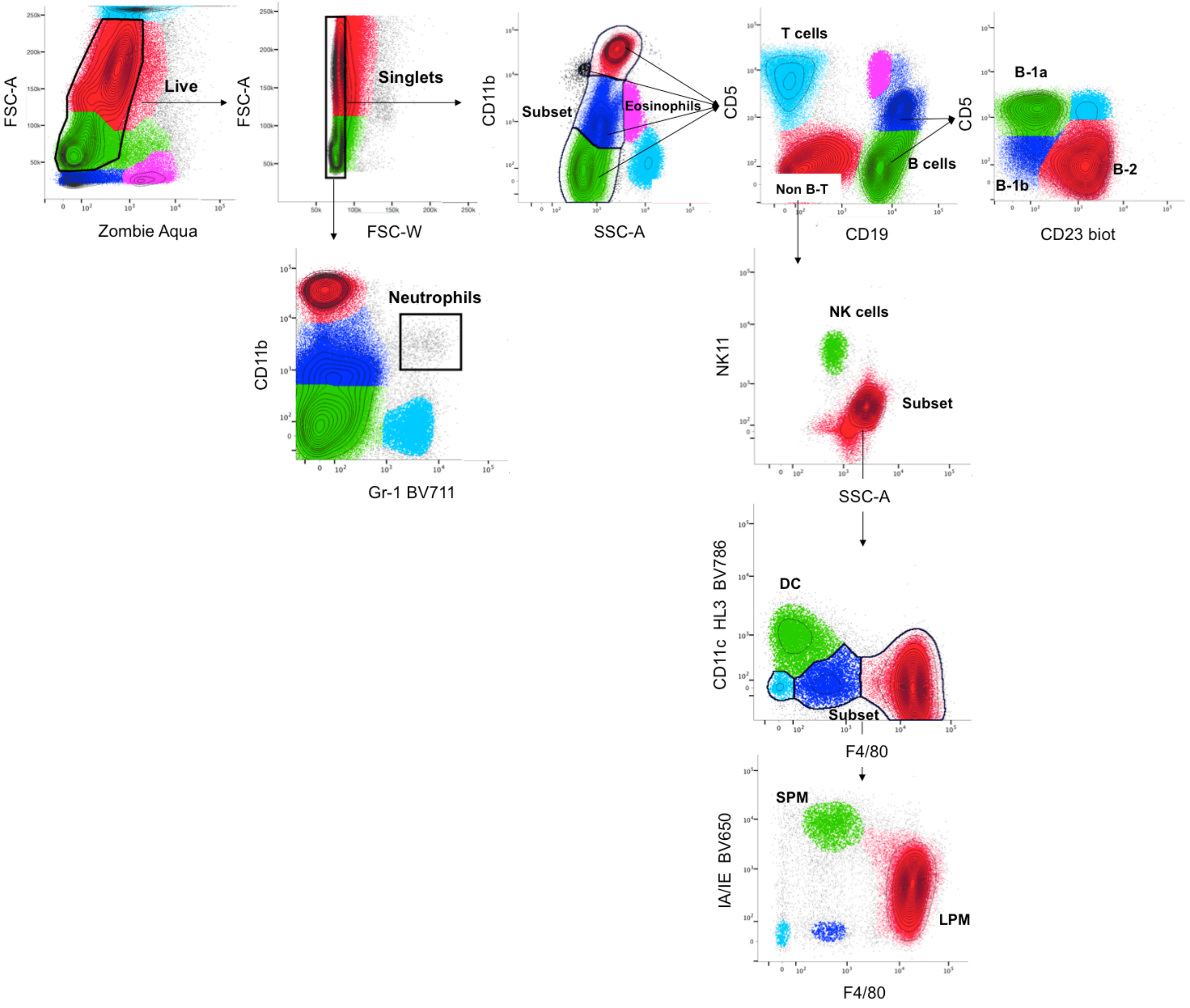

Supplementary Figure 5. Conventional gating strategy that we used to identify well-known subsets of mouse peritoneal (PerC) cells with user guided clustering.

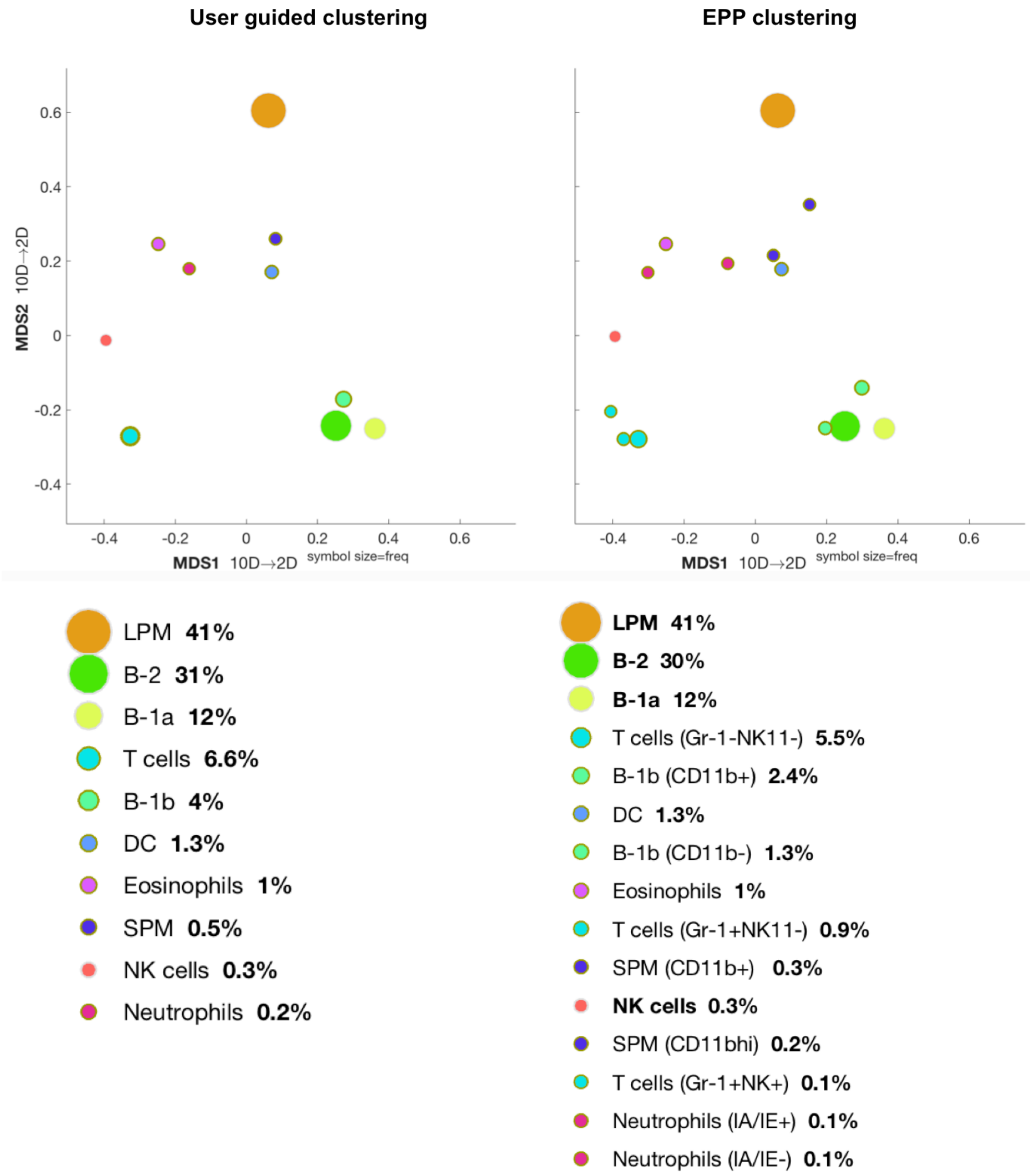

Supplementary Figure 6. SIC pipeline applied to mouse peritoneal cavity flow cytometry sample stained with different (than Figure 5a) antibody panel. Comparison of user guided and fully automated clustering outcomes for BALB/c mouse

PerC sample, only matched subsets are displayed. This dataset is available at https://flowrepository.org/id/RvFrvyOIlCe8Fbl8t3weWJvjUSh3CUQxkpwa4SPWgrrJRiBwiVSUEosPSreKYXuE

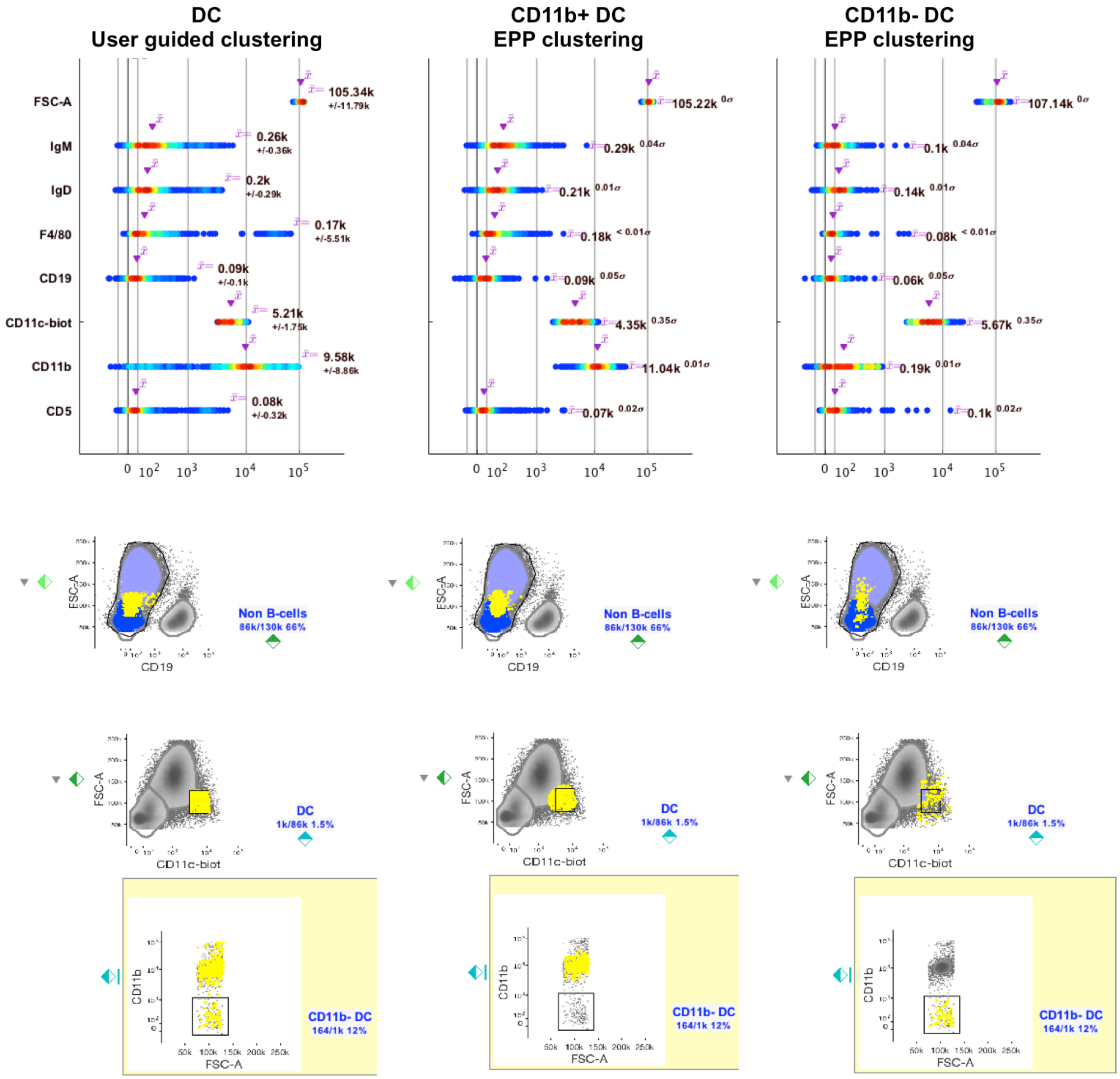

Supplementary Figure 7. Using the same set of parameters as in the manual gating strategy, SIC pipeline identified split in DC subset. "Pathfinder" tool provided by AutoGate (http://cytogenie.org/path-finder) was used to show the staining/scatter signal on measured parameters for selected cells. Pathfinder depicts each parameter with a horizontal bar that uses pseudocolor convention to show where the staining/scatter signal is most intense. Each horizontal bar is accompanied by the median value for this bar. AutoGate provides an ability to do backgating via its

“highlighter” feature. Cell population location on the gating tree (or any other pair of dimensions) can be automatically highlighted. For example, here we did backgating by automatically highlighting (shown in yellow) gating tree position of the dendritic cells (DC) identified with the fully automated and user-guided approaches.

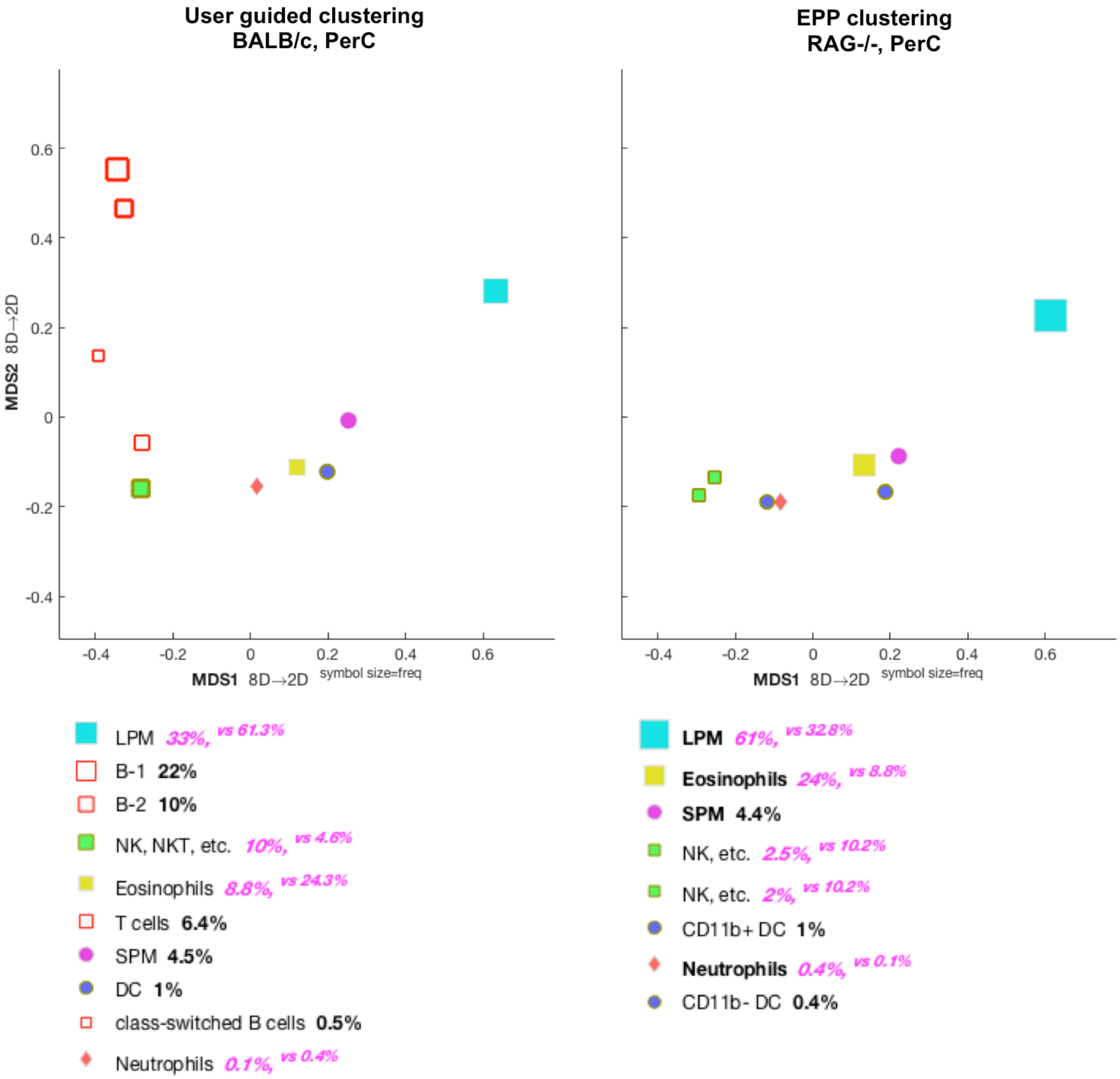

Supplementary Figure 8. SIC pipeline detected lack of lymphocyte compartment in RAG-/- mouse. Unmatched subsets (T cells, B-1, B-2 and class-switched B cells) presented as red squares. Cell subsets with medians located more than two standard deviations at least in one dimension are presented as diamond shape. Filled square shape highlights matched cell subsets with more than three percent difference in relative cell frequency between them. Each sample (BALB/c PerC and RAG$^{-/-}$ PerC) contains 200,000 cells.

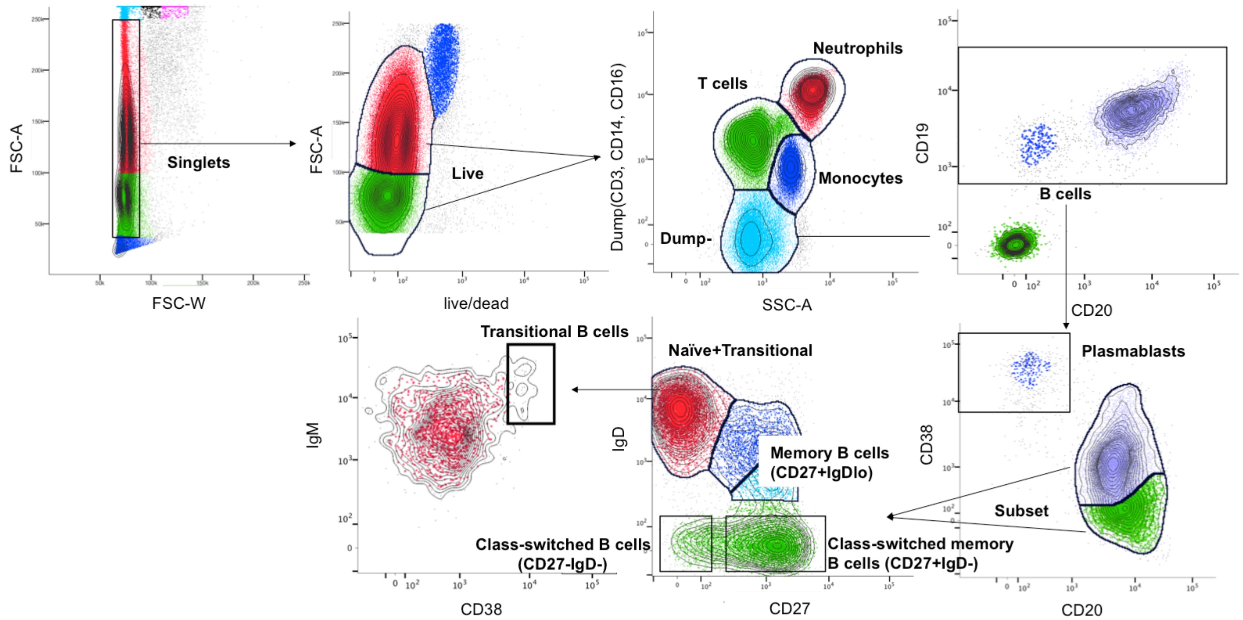

Supplementary Figure 9. Conventional (user guided) gating strategy that we used to identify well-known human B cell subsets (naive, memory, plasmablasts, etc.) with user guided clustering.

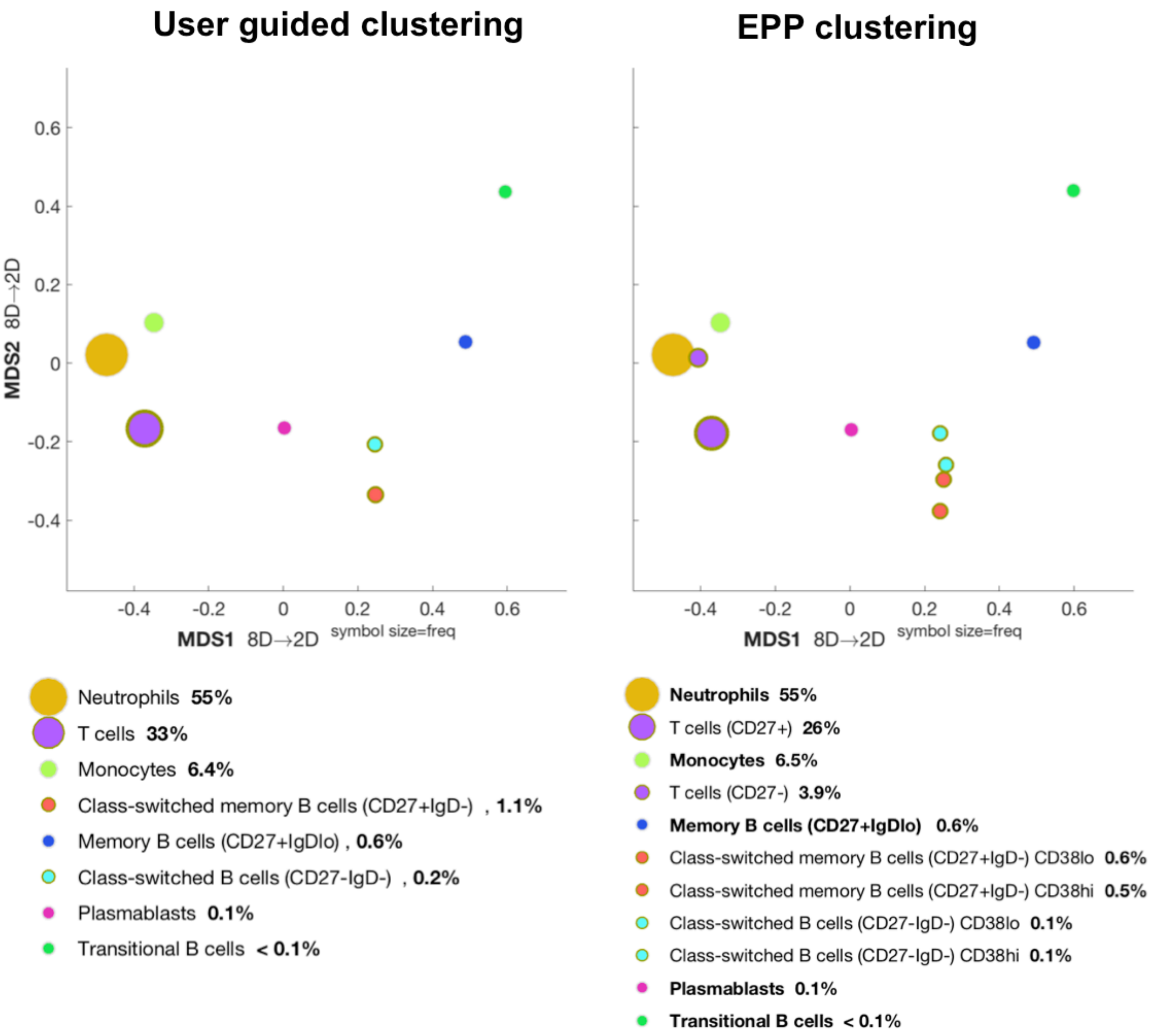

Supplementary Figure 10. SIC pipeline consistently identifies the main myeloid and lymphoid cell subsets in the human peripheral blood stained with the same panel of surface markers. Results are shown for a different (rather than one presented on Figure 6) human peripheral blood sample (~440k live singlets), and only the matched subsets are displayed.

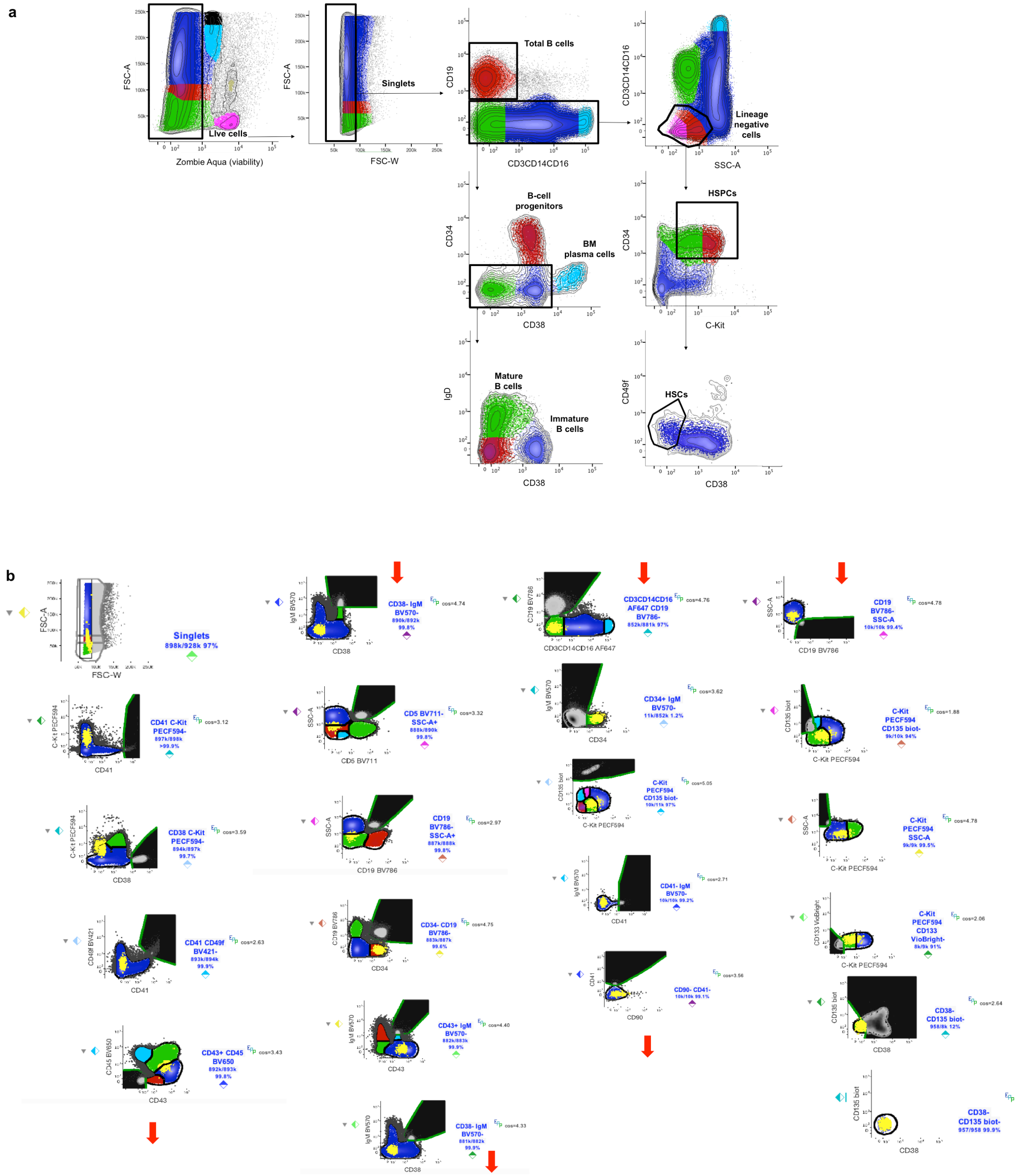

Supplementary Figure 11. Gating strategies used to identify HSCs subset. Panel a. Conventional gating strategy used in user guided clustering. **Panel b.** Gating strategy established by the EPP with the use of all markers available in the staining panel.

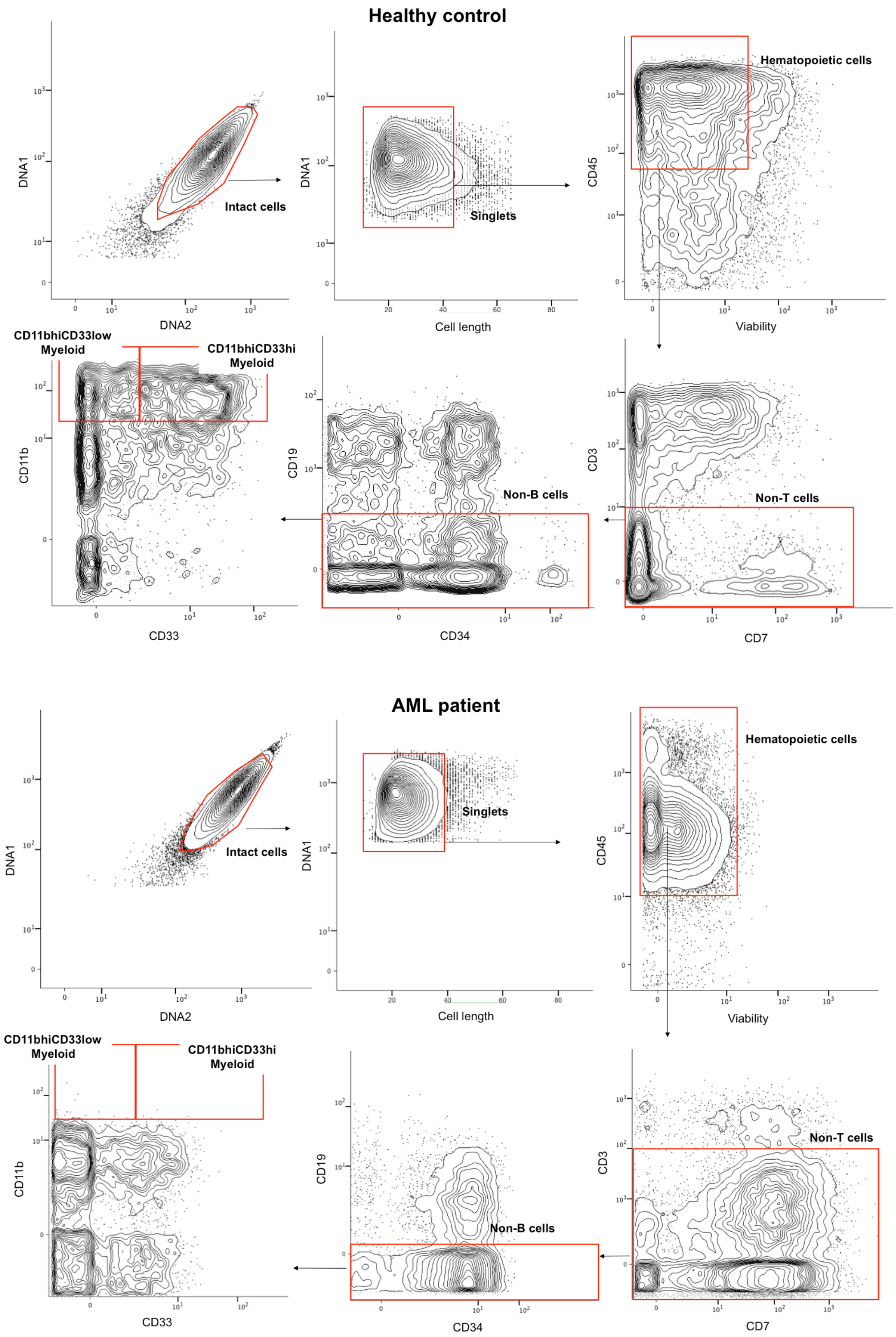

Healthy control
Intact cells
DNA1
DNA2
Singlets
Cell length
Hematopoietic cells
CD45
Viability
CD11bhiCD33low
Myeloid
CD11bhiCD33hi
Myeloid
CD11b
CD33
Non-B cells
CD19
CD34
Non-T cells
CD3
CD7
AML patient
Intact cells
DNA1
DNA2
Singlets
Cell length
Hematopoietic cells
CD45
Viability
CD11bhiCD33low
Myeloid
CD11bhiCD33hi
Myeloid
CD11b
CD33
Non-B cells
CD19
CD34
Non-T cells
CD3
CD7

Supplementary Figure 12. Gating strategy that we used to identify $CD11b^{hi}CD33^{low}$ and $CD11b^{hi}CD33^{hi}$ myeloid cell subsets in healthy controls and AML patients with the user guided clustering.

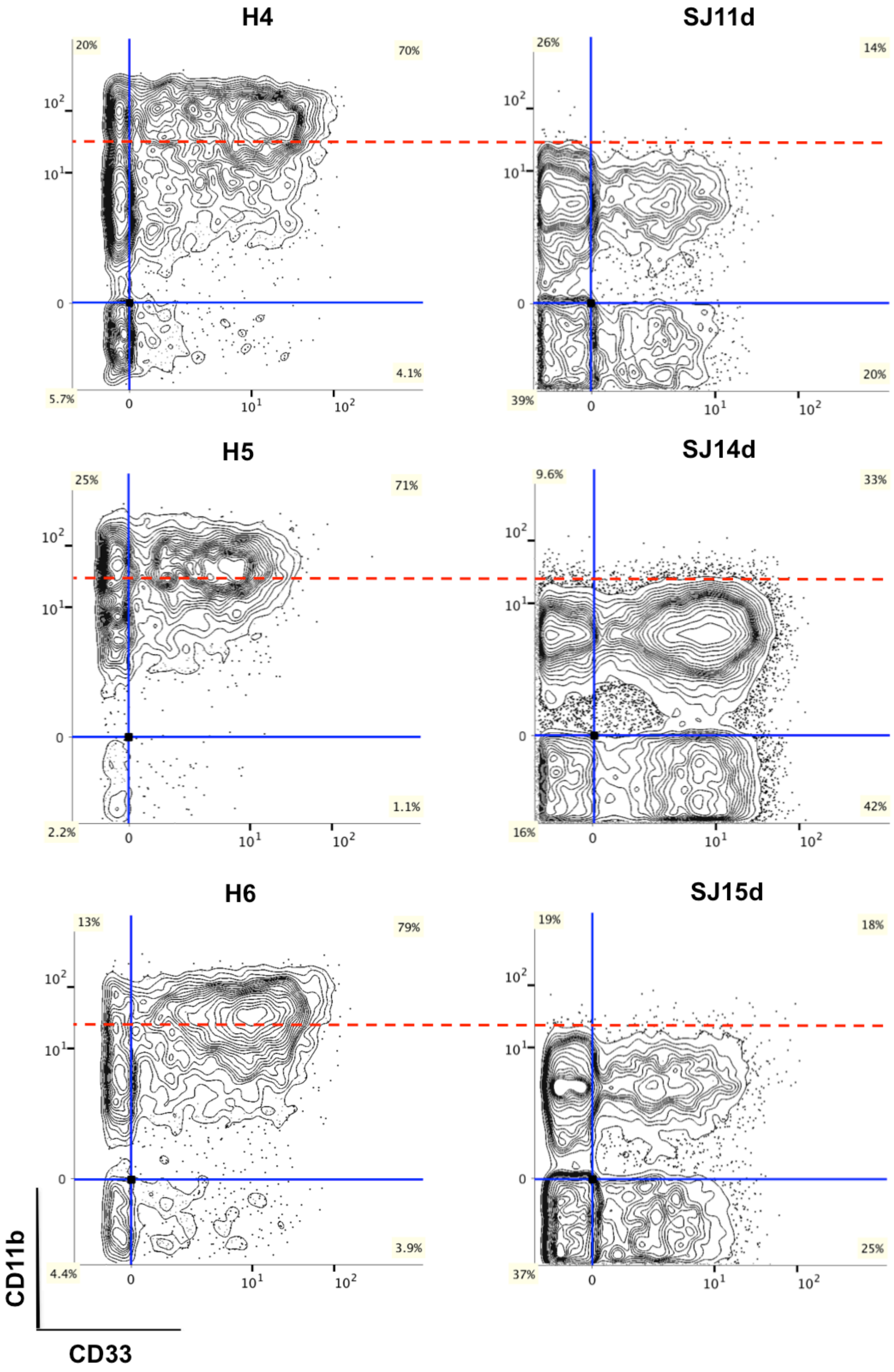

H4
SJ11d
H5
SJ14d
H6
SJ15d
20%
70%
4.1%
5.7%
26%
14%
20%
39%
25%
71%
1.1%
2.2%
9.6%
33%
42%
16%
13%
79%
3.9%
4.4%
19%
18%
25%
37%
CD11b
CD33

Supplementary Figure 13. The CD33+CD11b+ cell population shows significant difference between healthy (H4, H5, H6) and AML (SJ11d, SJ14d, SJ15d) samples.

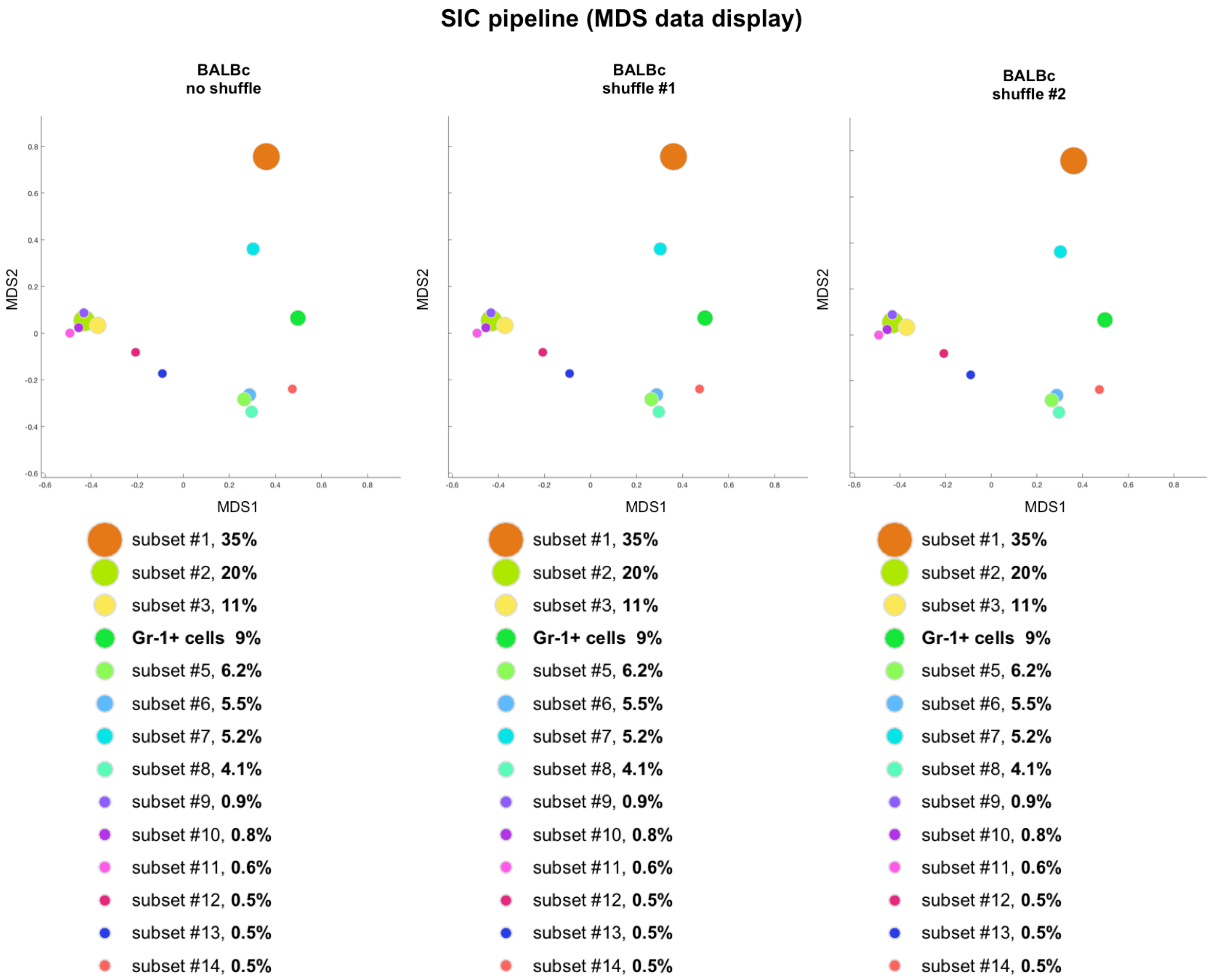

Supplementary Figure 14. SIC pipeline yields reproducible results (with MDS data display). We used the BALBc mouse dataset presented in Supplementary Table 2 to compare performance of SIC (QF-tree display is presented on Supplementary Figure 15), t-SNE and SPADE (Supplementary Figure 16) pipelines. We analyzed each sample (“BALBc no shuffle”, “BALBc shuffle #1”, “BALBc shuffle #2”) independently with exactly the same input parameters. Essentially, “BALBc no shuffle”, “BALBc shuffle #1”, “BALBc shuffle #2” contain exactly the same data but the order of events in “BALBc shuffle #1”, “BALBc shuffle #2” were randomly shuffled. For illustration purposes we picked Gr-1+IgM- cells as a target population and named it “Gr-1+ cells”.

Data were compensated and Logicle transformed with AutoGate utilities (www.cytogenie.com).

SIC pipeline (QF tree data display)

BALBc no shuffle

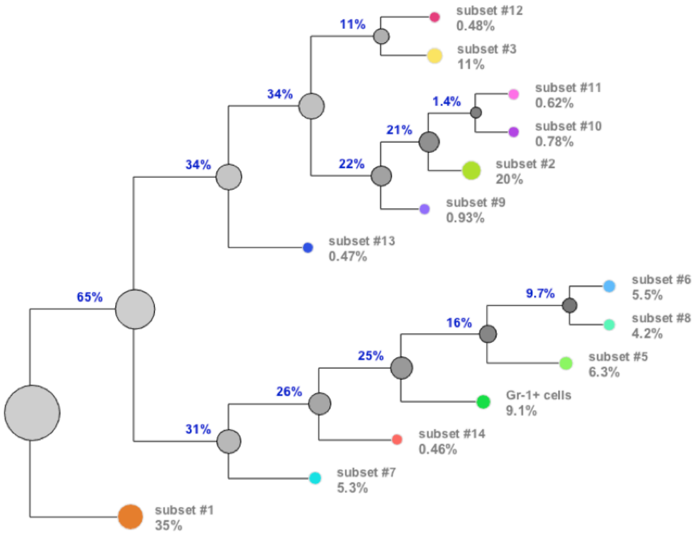

BALBc shuffle #1

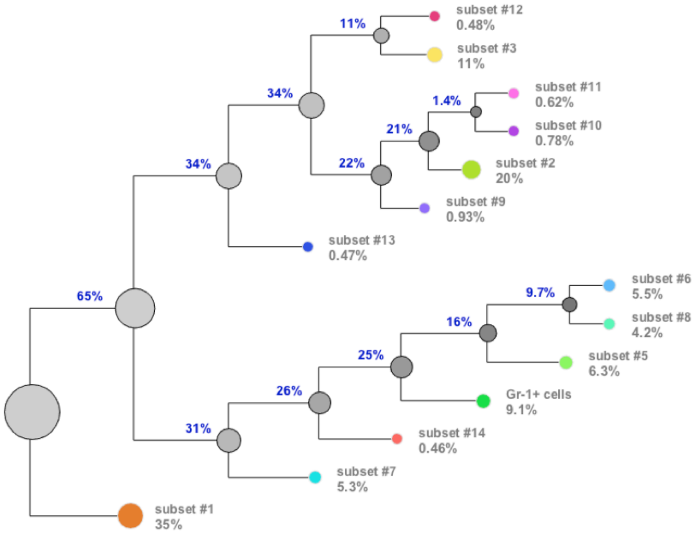

BALBc shuffle #2

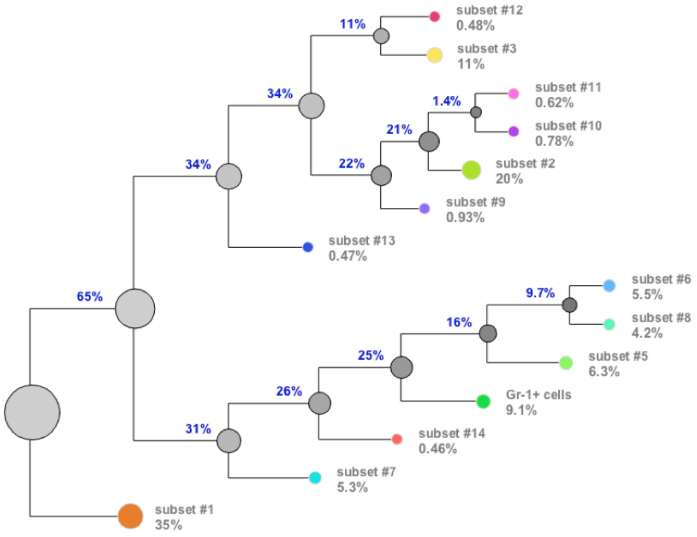

Supplementary Figure 15. SIC pipeline yields reproducible results (with QF-tree data display). Data source is discussed in Supplementary Figure 14 legend.

a

t-SNE

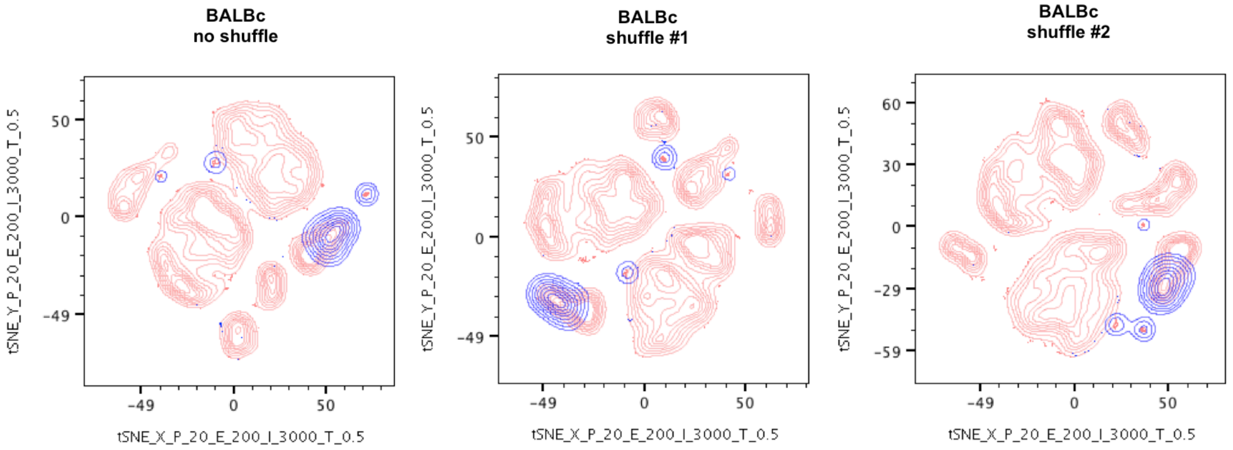

	Subset Name	Count
	Gr-1+ cells	517
	Ungated	10000

b

SPADE

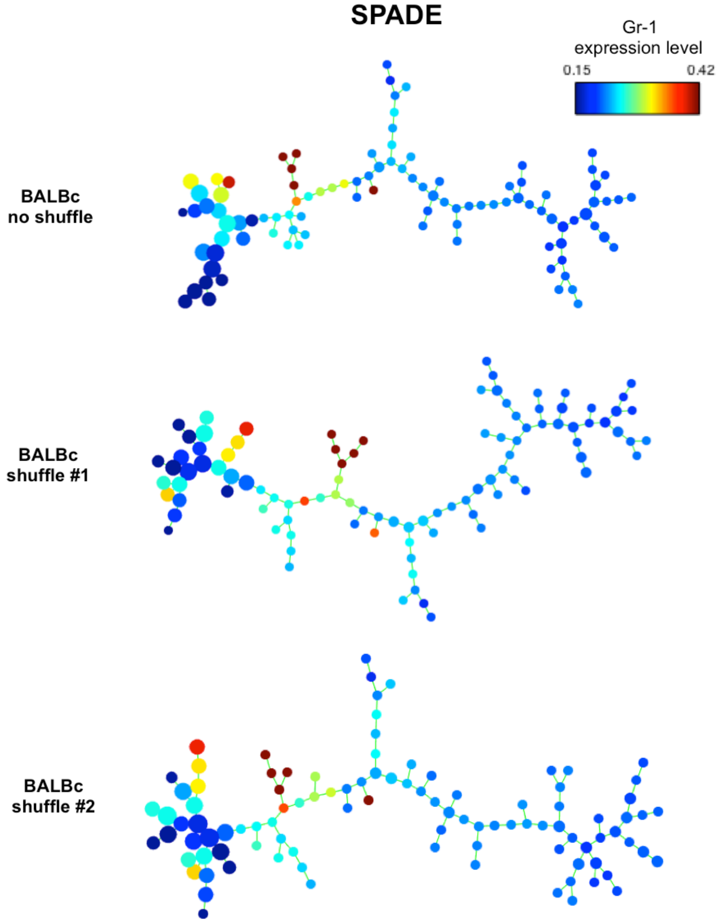

Supplementary Figure 16. Commonly used cluster identification and/or visualization pipelines (t-SNE and SPADE) may yield irreproducible results. Data source is discussed in Supplementary Figure 14 legend. Due to stochastic nature of t-SNE and SPADE algorithms Gr-1+ cells position, relative to itself and other cell populations, was not reproduced between “BALBc no shuffle”, “BALBc shuffle #1”, “BALBc shuffle #2” samples.

Here we used t-SNE plugin provided by FlowJo v10 and used the default input parameters except for the number of iterations (we used maximum allowed number of iterations, 3000, to make sure that t-SNE algorithm has converged). We used SPADE v3.0 and followed the guidelines for input parameters choice provided at http://pengqiu.gatech.edu/software/SPADE/ .

Supplementary Tables

Sample id	X-shift (angular distance)	Rphenograph	ClusterX	DensVM	flowMeans	EPP
1	3	2	3	3	2	2
2	3	2	2	2	2	2
3	5	2	4	5	2	2
4	3	2	4	5	2	2
5	4	2	3	4	2	2
6	9	2	3	5	2	2
7	5	2	3	3	2	2
8	4	2	4	6	2	2
9	2	2	4	5	2	2
10	7	2	2	2	2	2

Supplementary Table 1. Commonly used high dimensional methods may report populations that do not exist. We simulated a mixture of two 20D Gaussian distributions with unit variance in each dimension and the following means: M1: [0 0 0 0 0 0 0 0 0 0 0 0 0 0 0 0 0 0 0 0], M2: [2.3 2.3 2.3 2 2 2 1 1 2.3 2.3 2 3 5 1 3 2.3 2 4 5 2]. Each Gaussian distribution consists of 1000 events. The simulations were repeated ten times and the table gives the number of clusters reported for each simulation.

Sample		X-shift (angular distance)	Rphenograph	ClusterX	DensVM	flowMeans	EPP	DBM*
BALBc	no shuffle	49	14	25	8	7	14	125
	shuffle #1	69	14	26	15	7	14	125
	shuffle #2	82	13	24	11	6	14	125
C57	no shuffle	63	15	28	13	3	11	123
	shuffle #1	48	15	27	15	3	11	123
	shuffle #2	81	14	24	10	3	11	123
RAG -/-	no shuffle	78	14	26	8	5	6	101
	shuffle #1	67	14	24	6	4	6	101
	shuffle #2	50	12	25	9	4	6	101

Supplementary Table 2. Commonly used high-dimensional clustering methods may yield irreproducible results. The table shows the number of clusters identified by the seven distinct clustering algorithms applied to samples and to random shuffles (events order is shuffled randomly in each file) of these samples. We tested algorithms that work directly on the Hi-D data (X-shift [10,11], Rphenograph [23] and flowMeans [24]), algorithms that are applied to the t-SNE embedded map (ClusterX [23] and DensVM [23]), and sequential user-guided (DBM [25]) and fully automated (EPP, www.cytogenie.org) clustering algorithm. We used a 10-color dataset (10-color + Side and Forward Scatter, dataset is available at https://flowrepository.org/id/FR-FCM-ZZJF). Data were compensated, Logicle transformed and pre-gated for live singlets using AutoGate (www.cytogenie.org). Data were downsampled using FlowJo v10. We used the default input parameters provided by each clustering algorithm but omitted the data transformation since the data were already Logicle transformed. * Since DBM is a 2D

sequential clusterering algorithm, here we report the sum of clusters from all 2D projections. However, total number of clusters on each 2D projection was the same among samples and their shuffles.

Sample		X-shift (angular distance)	Rphenograph	ClusterX	DensVM	flowMeans
**BALBc** random 2D projection #1	no shuffle	14	27	50	9	2
	shuffle #1	16	28	62	9	2
	shuffle #2	18	28	59	12	2
**BALBc** random 2D projection #2	no shuffle	18	32	54	6	2
	shuffle #1	18	29	54	17	2
	shuffle #2	16	29	63	11	2
**C57** random 2D projection #1	no shuffle	18	29	53	5	3
	shuffle #1	18	29	62	7	3
	shuffle #2	16	27	78	8	3
**C57** random 2D projection #2	no shuffle	19	28	59	10	2
	shuffle #1	16	28	54	20	2
	shuffle #2	16	26	45	16	2
**RAG -/-** random 2D projection #1	no shuffle	15	28	62	10	3
	shuffle #1	17	30	63	6	2
	shuffle #2	14	29	65	5	2
**RAG -/-** random 2D projection #2	no shuffle	17	28	56	9	2
	shuffle #1	13	27	58	6	2
	shuffle #2	15	27	53	6	2

Supplementary Table 3. The severity of a randomization problem diminishes in low dimensional space. From each set of thirty- six 2D projections, we randomly picked two 2D projections and ran clustering algorithms for each 2D projection and for two random shuffles of this 2D projection. The table shows the number of clusters identified by the clustering algorithms discussed in Supplementary Table 2 legend.

Group ID	**1**	**2**	**3**	**4**
**1**	-	3.75	7.77	7.36
**2**		-	5.01	3.74
**3**			-	2.90
**4**				-

Supplementary Table 4. Matrix of distances between groups' medians in Sample B.

Supplementary Note 1

Curse of dimensionality in flow/mass cytometry data analysis

The ability to identify populations (clusters) of objects/subjects based on their similarity in multiple measured dimensions is still one of the most pressing unsolved problems in extracting meaning from “big” datasets such as those collected in industrial, medical, biological, business and other settings. Although not commonly recognized, current medical research and practice are limited by reliance on subjective manual analyses of flow cytometry datasets.

Such datasets, typically collected for multiple samples, are commonly high-dimensional (aka multi-parameter or Hi-D) measurements representing 12 or more dimensions recorded for up to several million cells. Most users still analyze such flow data manually using analysis packages such as Flowjo (http://www.flowjo.com), DiVa (http://www.bdbiosciences.com) and FCS Express (www.denovosoftware.com). The optional manual gating features in these analysis packages sequentially display data for pairs of dimensions and allow users to draw gating boundaries around subjectively-identified subsets and to then select one or more these subsets for the next analysis round. Users then repeat this (recursive) subset display and subset selection process until all subsets of interest have been identified.

Although slow and cumbersome, these manual gating “Projection Pursuit” approaches work quite well, at least in the hands of skilled operators. In fact, much of what is known about stem cells, blood cells, and diseases such as leukemia and AIDS relies on flow cytometry data analyzed with these methods. Additionally, these methods are also

central to a wide variety of clinical assays that provide life and death information on a daily basis. Thus, blood samples collected in a clinical setting contain cells whose individual properties, measured by Hi-D flow cytometry, enable their assignment to subsets (e.g., leukemic cells, lymphocytes, granulocytes), which are defined by distinctive combinations of dimension values that collectively reflect levels of surface or internal markers expressed on/in the individual cells in individual subsets.

Over the years, the number of identifiable blood cell subsets has increased substantially, as has the medical importance of being able to resolve them. However, resolution of these subsets with the available manual tools is by no means routine. In fact, because current analysis methods ultimately rely on user skills to manually define subset boundaries and other properties, identification and quantitation is still more appropriately recognized as an art rather than a science.

Automating this data analysis process is clearly desirable. Thus, several groups have recently developed intensive computational approaches aimed at simultaneously identifying the subsets (clusters) within a given Hi-D dataset [1]. However, while these attempts at Hi-D clustering methods are well motivated from a biomedical and user functionality point of view, they are perforce highly sensitive to compromise by what statisticians refer to as “the curse of dimensionality” [2,3,4].

The curse of dimensionality is a well-known statistical problem that compromises both statistical validity and computational performance of Hi-D clustering methods [2,3]. In flow and mass cytometry, however, the impact of the “curse” has only recently been recognized, and then only as a computational problem that mainly affects the

performance of Projection Pursuit approaches [5], i.e., it is computationally expensive to use two-dimensional plots to inspect all pairs of markers in Hi-D flow cytometry dataset. However, even when this problem is resolved (i.e., as we show here, by applying an informed, fully automated Projection Pursuit approach), there exists a key statistical aspect of the curse of dimensionality that compromises the validity of Hi-D clustering outcomes.

Not yet properly recognized in the flow/mass cytometry field, this statistical problem clearly cautions mitigates against the use of Hi-D clustering methods for flow/mass cytometry data analysis. This problem arises from the marked increase in statistical uncertainty that occurs as the number of dimensions for which data is being considered increases. In essence, as the number of dimensions increases, any cube with a given side length progressively contains a shrinking fraction of the observations (Supplementary Figure 1). In fact, this fraction shrinks so rapidly that even when trying to find subsets in a dataset taken for a moderate number of dimensions (<5), the data may be too sparsely distributed to yield reliable results. Basically, as D.W. Scott shows in Chapter 7.2.1 of Multivariate Density Estimation - Theory, Practice and Visualization [6], in order to reach a given accuracy for typical Hi-D methods, the sample size has to increase exponentially with dimension. Table 4.2 in [4] shows that in order to get the same accuracy in density estimation, in a 10-dimensional setting the sample size needs to be 40,000 times larger than in a 2-dimensional setting.

Here, we present some simple and concrete examples that demonstrate this point. In addition, we show how the curse of dimensionality leads to invalid conclusions by

commonly used Hi-D flow/mass cytometry data clustering methods (e.g., DBSCAN [7], flowMeans [8,9], X-shift [10,11]).

The curse of dimensionality also impacts the computational time required to analyze the data using Hi-D clustering methods. In essence, optimization problems that arise in fitting multi-parameter models become progressively more complex as the number of dimensions increases [12], making the time required for the analyses progressively more formidable. Parallelizing the computation can help, but the computation time still tends to become prohibitive when higher dimensional datasets are analyzed. These considerations led Qian et al [13] to caution designers of model-based clustering methods for flow data analysis to beware of the curse of dimensionality.

Importantly, however, the curse of dimensionality is not restricted to affecting model-based clustering (or cluster matching). It can also affect the accuracy and reliability of any simultaneous clustering method, and typically will do so.

Supplementary Note 2

The curse of dimensionality in clustering

There are multiple well-recognized data analysis problems shown to be due to the curse of dimensionality in domains such as data mining, machine learning, clustering, etc. Here we describe three main manifestations of this curse that compromise the clustering of data in Hi-D flow/mass cytometry:

> Data becomes increasingly sparsely distributed as the number of dimensions increases (Supplementary Figure 1);

Definitions of density and distance between points become increasingly meaningless (Supplementary Figure 1b and Supplementary Figure 2);

Fitting a mathematical model to a dataset becomes infeasible because the number of combinations of possible parameters to be considered increases dramatically as the number of dimensions increases above three or four. Indeed, as Qian et al. [13] point out, “It remains an open question if model-based approaches can solve the high-dimensional clustering problem… [since] the populations may not follow the distributional models when they become sparse in the high-dimensional space due to the “curse of dimensionality”.”

The above manifestations of the curse of dimensionality directly apply to (but are not limited to) the following four general approaches to clustering that rely on density and/or distance estimation, or the fitting of mathematical models:

Hierarchy algorithms that create a hierarchical decomposition of the dataset using some criteria (e.g., k-nearest neighbor (kNN) density estimation in X-shift algorithm [10]; L1 distance metric in SPADE [14])

Partitioning algorithms that construct various partitions and then evaluate them by some criteria (e.g., k-means clustering in flowMeans [8])

Density-based algorithms that are based on connectivity and density functions (e.g., FLOCK [13])

Model-based algorithms in which a model is hypothesized for each of the clusters in order to find the best fit of that model (e.g., flowClust [15], FLAME [16], SWIFT [17])

Thus, as the above-mentioned approaches indicate that, data sparsity, loss of reliability in distance measurements, and major complexities of fitting are critical limitations for clustering of Hi-D flow/mass cytometry datasets.

In Hi-D flow/mass cytometry datasets, most cells typically express only a fraction of the markers (~2-6) used in a given Hi-D (e.g., 20-color) staining panel. Furthermore, a huge sample size is required to locate a subset of cells that are positive for only a few of the markers detected by the Hi-D panel (see legend for Supplementary Figure 1). This is a critical limitation that prohibits clustering flow/mass cytometry datasets in Hi-D space.

Indeed, Supplementary Figure 3 illustrates how the curse of dimensionality impacts the conclusions of the commonly used Hi-D flow/mass cytometry clustering methods (DBSCAN [7], flowMeans [8, 9], and X-shift [10,11]). Supplementary Figure 3a shows a plot of the first two coordinates of a simulated mixture of two 20D Gaussian distributions whose means differ in only those 2 coordinates. The two populations appear to be clearly separable in this projection. Indeed, commonly used clustering methods detect the presence of two populations and separate them well when applied in this 2D situation (Supplementary Figure 3b). However, these methods fail to detect the presence of two populations when applied in the Hi-D 20D setting (Supplementary Figure 3c) due to the fundamentally larger statistical uncertainties in the 20D situation.

Even more alarming, the Hi-D clustering methods may report populations that do not exist (Supplementary Table 1). This erroneous overestimation of the number of populations is again partially due to the curse of dimensionality, as the sparsity of data in high dimensional space will make it likely that, just by chance, multiple clusters

appear in various parts of the space. It is difficult to properly calibrate statistical procedures to protect against this error, as the results in Supplementary Table 1 show. Clearly, such an erroneous finding of spurious populations would be a serious scientific error. As we showed before [3] the curse of dimensionality may cause not only "under/overclustering" of the data but also can cause failure to define cluster boundaries (see Figure 1b in [3], X-shift example).

In an attempt to solve this problem, several data dimensionality-reduction approaches, e.g., as t-SNE [18], were recently introduced as a preprocessing step in anticipation of data clustering. Though t-SNE is subject to intensive parameter tuning [19], it is a powerful visualization tool for Hi-D data with intrinsically low dimensional structure, i.e., when N dimensional data can be closely approximated by some combination of $n<<N$ dimensions. However, in cases where the intrinsic dimensionality is high, or the data points sit on a highly varying manifold, t-SNE is known to perform poorly, since it's most basic assumption - local linearity on the manifold - is violated [18].

This problem, referred as “the curse of intrinstic dimensionality”, compromises dimension-reduction methods that are designed to work in situations in which the nominal dimension of the data is high, but the data are restricted to a much lower dimensional manifold. As stated by Levina et al. [20]: “There is a consensus in the high-dimensional data analysis community that the only reason any methods work in very high dimensions is that, in fact, the data are not truly high-dimensional. Rather, they are embedded in a high-dimensional space, but can be efficiently summarized in a space of a much lower dimension, such as a nonlinear manifold. Then one can reduce dimension

without losing much information for many types of real-life high-dimensional data, such as images, and avoid many of the "curses of dimensionality" ".

Quite often, data that are observed in a high-dimensional space follow a structure that is lower-dimensional. For example, the swiss roll (http://people.cs.uchicago.edu/~dinoj/manifold/swissroll.html) is a 2-dimensional sheet in the 3-dimensional space. If data fall on such a lower-dimensional structure, then the problems associated with the curse of dimensionality may be reduced, provided there is an analysis method that can find this lower-dimensional structure and exploit it [21]. Therefore, it is of interest to investigate whether such a structure typically exists in flow cytometry data.

The dimensionality of such a structure is called the intrinsic dimensionality of the data (for a rigorous definition, see [20]). Informally, the intrinsic dimensionality is the number of variables needed to represent the structure in the data, i.e. the number of variables needed to parametrize the structure, under the provision that the data follow this structure apart from some small deviations due to noise, measurement error etc. For example, in the "swiss roll" example, a sheet in 3D space is two-dimensional, hence the intrinsic dimensionality of data that fall in a close vicinity of such a sheet is two, even though the dimension of the ambient space is three.

To find out whether, in flow cytometry, the intrinsic dimensionality of a typical dataset is much lower than the measured number of dimensions, we used the Maximum Likelihood Estimation of Intrinsic Dimension (MLE) method proposed by Levina et al. [20,22]. MLE revealed seven intrinsic dimensions for the 17-parameter flow cytometry sample (15-color + Side and Forward Scatter, dataset is available at

https://flowrepository.org/id/RvFrUV5PwhMD3N6eyLM3zyo5xFuReZOfa3FYAY2DvSnv DJaGzto0QH0kPH7Pfkgg) and five intrinsic dimensions for the 12-parameter flow cytometry sample (10-color + Side and Forward Scatter, dataset is available at https://flowrepository.org/id/FR-FCM-ZZJF). However, as we have shown above (Supplementary Figure 1), even three dimensions can be problematical for flow/mass cytometry data, and the severity of the curse of dimensionality problem increases sharply thereafter.

In high-dimensional spaces, effects attributed to the "curse of dimensionality" can hinder reliable statistical inference in many ways. For example, clustering and dimensionality-reduction algorithms that use randomization for inference (e.g., t-SNE has the objective function which is minimized using a gradient descent optimization that is initiated randomly) have become progressively unreliable with the increasing number of dimensions and can report irreproducible results (Supplementary Figure 4).

Here we demonstrate how this randomization problem gets worse with the number of dimensions (compare Supplementary Table 2 and Supplementary Table 3) and leads to irreproducible clustering results.

Supplementary Note 3

SIC pipeline performance and scaling

The software under discussion here is the AutoGate implementation of the SIC pipeline.

The pipeline is mostly programmed in MatLab.

The performance of the pipeline steps are as follows:

EPP

The EPP method will be published separately (manuscript is in preparation) but we summarize it here to help the readers understanding. EPP is designed to find subsets based on phenotyping markers and scatter parameters but not based on stimulation or intra-cellular markers. Performance impacts come from the number of dimensions, cells and clusters per 2D projection:

Dimensions: each additional dimension increases performance quadratically by expanding the number of 2D projections. 11D has 55 2D pairings, 12D has 66,13D has 78... 30D has 435, 31 requires has 465.

Cells: increase in cell counts has a linear impact on the cost of creating DBM's density grid. Most of the processing after this remains the same since it works with the grid. There is also linear cost related to memory use.

Clusters per 2D projection: each additional cluster that DBM finds impacts performance

Combinatorically for EPP's contiguity check. EPP checks all 2-way splits of a 2D projection's clusters to see if the clusters with each side of the split are contiguous with each other.

Near-combinatorically for computing the separatrix of contiguous splits.

The combinatoric cost can be illustrated with a 4-cluster projection, this entails the following combinations:

1 + (2 3 4)

(1 2) + (3 4)

(1 3) + (2 4)

(1 4) + (2 3)

(1 2 3) + 4

(1 2 4) + 3

(1 3 4) + 2

QFMatch

Performance impacts come from the number of dimensions, cells, cell-overlap and merge candidates.

Dimensions: unlike the EPP step, the QFMatch step handles additional dimensions in a linear manner and only during the initial sub task of adaptive binning as well as the sub task of calculating distances during quadratic form dissimilarity computation.

Cells: this impacts performance when it increases the total adaptive bin count for a subset. AutoGate uses slower non-vectorized programming modules for subsets with more than 200,000 cells because it risks running out of memory if it uses fast vectorized programming. Vectorized programming uses matrices without “for loops” for all terms of the quadratic form dissimilarity formula instead of scalars and for loops. In MatLab this has been observed to improve performance over even a C implementation with scalars. MatLab r2017a may use the SIMD operations of the Intel hardware for vectorized operations. When larger subsets exist, the AutoGate user can choose to ignore the slower comparison operation for large subsets effectively removing the particular subset from the analysis.

Cell overlap: This can impact the quality of performance more than the time of performance. Why? When quadratic form-based match compares 2

groups of subsets and subsets in either group have cells that other subsets in the same group also have (this can happen when manual gating is used to identify cell subsets), then the QFMatch has been observed to mislead. Thus, AutoGate does a check for overlap and warns the user of this situation.

Merge candidates: an increase in merge candidates impacts quadratic form-based match combinatorically. Its' impact on quadratic form-based match can be costlier than any other impact in the entire pipeline. A high number of merge candidates happens when, between the 2 groups of subsets being matched, there is one subset in one group that matches best with 2 or more subsets in the other group. To resolve the best match, the algorithm requires choosing the best quadratic form dissimilarity measure for every combination of merge candidates including single unmerged candidates. This likelihood of long running combinatorics increases as the difference in number of subsets between groups differs. E.g., matching a group of 8 subsets with a group of 12 subsets is less likely to suffer long merge testing than matching a group of 8 subsets with a group of 52 subsets. A subset "best match" is only ineligible for merge testing if at least one of the parameters has a median more than four standard deviation units different when using normal distributed data (logicle for FACS and Log10 for CYTOF due to well known issues with dull signal detection).

The impact of large merging tasks is addressed by the:

Software (AutoGate) avoiding uncertain computer-frozen impressions through detailed progress reports that allow the user to cancel (no computer lock up) without data loss consequences.

User halting the matching:

Cancelling the entire computation

De-selecting merge testing for subsets in which they are less interested (and then continuing the computation). If a user selects to ignore merge testing for one or more subsets then this action only alters the compute time and not the correctness of the matching results for the other subsets in which the user remains interested.

User defining additional known subsets when and if the smaller group of subsets being matched contains non EPP subsets. The additional subsets need only be density clusters for cells that are not already contained by the group's subsets. Groups of EPP subsets cannot have this remedy since EPP addresses every cell. Thus, no remedy exists when the smaller match group is EPP subsets.

MDS

AutoGate does multi-dimensional scaling (MDS) of subset medians with MatLab's built-in function cmdscale. MDS is done strictly for the purpose of a quick visualization in conventional MatLab 2D plots. The operation of MDS is always faster than the cost of the visualization so not much needs to be reported on it here. Moreover, since AutoGate's MDS only considers subset medians, users are

encouraged to use the subsequent visualization for a quick report on quadratic form-based matching rather than a guide to the HiD relatedness of the subsets. For HiD relatedness the primary AutoGate visualization is its Phenogram which considers all of the data. Our manuscript refers to Phenograms as QF-tree.

QF-tree

AutoGate produces a visual dendrogram to express HiD relatedness. The non-visual processing defaults to the use of quadratic form dissimilarity metric on the adaptive binning of all of a subset's data plus Euclidean distances on the subset medians. AutoGate offers the user other distance/dissimilarity measures. The distance-only alternatives, however, consider medians and thus suffer from the same risk of under-informing as does AutoGate's MDS visualization. The majority of the QF-tree's computation cost is the non-visual processing. The visualization invokes MatLab's phytree function and then performs customizations on the visual objects output by phytree.

QF-tree performance is impacted by number of cells and subsets in a linear manner. The impacts are not major since there is no quadratic or combinatorial change in workload when input factors scale up or down. QF-trees have the risk of slowness for the same reasons as described previously with QFMatch: accelerating the quadratic form distance dissimilarity and Euclidean distance calculations with vectorized programming requires pre-allocating memory in amounts which increase exponentially with larger subsets. Thus, slower non vectorized programming must occur.

Specific examples of performance

Computers:

Laptop running macOS High Sierra (Computer 1), 2.9 GHz intel i9 processor with 6 cores+265K cache, 32 GB 2400 MHz DDR4 memory.

Laptop running Windows (Computer 2) 10, 2.6 GHz intel i7 processor with 2 cores + 512 cache, 16 GB DDR2 memory.

Samples:

This paper's conventional flow cytometry sample of mouse peritoneal cavity cells that investigates macrophages and B cells.

A conventional flow cytometry sample of 4 million events in human nasal tissue (not presented in this paper).

Pipeline cases:

Conventional flow sample on 127, 392 cells with dead, doublets and debris gated out:

EPP (using low clustering details) on 11 dimensions which produces 16 final subsets (i.e., last gate of 16 sequences) is resulted in the compute cost of 3 minutes on Computer 1 and 12 minutes on Computer 2.

QFMatch of above 16 EPP subsets to 14 subsets gated by expert is resulted in the compute cost of 4 seconds on Computer 1 and 6 seconds on Computer 2.

QF-tree on above 16 Epp subsets is resulted in the compute cost of 9 seconds on Computer 1 and 10 seconds on Computer 2.

Conventional flow sample on 3,161,339 cells with doublets gated out:

EPP (using low clustering details) on 11 dimensions which produces 21 subsets is resulted in the compute cost of 8 minutes on Computer 1 and 24 minutes on Computer 2.

QFMatch

On above 21 subsets is resulted in the compute cost of 24 seconds on Computer 1 and 41 seconds on Computer 2.

On 14 subsets from a 2nd Epp run with less parameter reuse on the same data is resulted in the compute cost of 20 seconds on Computer 1 and 35 seconds on Computer 2. Note that this 2nd Epp run avoids dimensions used in parent subsets while the 1st Epp does not avoid reuses. Otherwise the 2nd EPP run is identical to the 1st in every respect. The difference means that for the 2nd run, at each level of the gating hierarchy Epp does NOT consider parameter pairings if at least one or the pair's parameters is already used in a prior level of the gating hierarchy starting at the level where Epp starts running. The 2nd run is built to test quadratic form-based matching for large subsets. With this data set some subsets are too large for vectorization and thus AutoGate uses the slower non vectorized JAVA.

QF-tree on above 21 EPP subsets is resulted in the compute cost of 54 seconds on Computer 1 and 54 seconds on Computer 2.

Current implementation of the SIC pipeline has yet to be optimized other than those sections which can be treated with MatLab vectorization optimizations. Future

optimization steps may include: refactoring the code to work (independently of the AutoGate desktop GUI on a server; in parallel threads or processes); converting to C or JAVA the portions of the pipelines which are either timing or memory constrained or both. Timing constraints are areas that cannot be vectorized in the MatLab grammar and thus need conversion to C or JAVA. With MatLab's 2017a version we have observed vectorizations to come close to, or in some cases surpass, the speed of C. This is best observed with data compensation where our MatLab vectorized implementation is slightly faster than the C implementation which uses for loops on scalars. Memory constraints are those vectorizations whose matrix/vector sizes exhaust physical memory with large data sets. Conversion to "for loopy" C or JAVA will be slower but not deplete memory.

Supplementary Methods

Updated DBM clustering algorithm

The original idea of the DBM clustering approach was described in our previous paper [25]. Here we introduce the simplified logic for this algorithm together with some recent corrections and additions.

Step 1. Represent a distribution on a grid and estimate the density in each grid point. During this step, we are creating a grid (2-dimensional in the current implementation) with associated weights that represent an approximation of the observed flow cytometry data. Then we use these weights to estimate the density surface. Thus, we bin the data on a grid y_m, consisting of M^2 points (typically M = 128 or 256, $m = (m_1, m_2) \in \{1, \dots, M\}^2$). Associated weights $\{w_m; m \in \{1, \dots, M\}^2\}$ and cell

density $\hat{f}(y_m)$ are computed at each grid point as described in our previous paper [25]. In the current implementation of DBM we use as bandwidth $h_j = h_{min,j}$ if $n>N_{min}$ and $h_j = h_{min,j}*(n/N_{min})^{(-1/6)}$ otherwise, where $h_{min,j} = ClusterDetailSetting*(max_i x_{ij} - min_i x_{ij})$ and $N_{min} = 5000$. By default $ClusterDetailSetting$ is 0.023 which we represent as "medium" cluster detail to the user. We offer 4 other alternative detail levels: "very high" where $h_{min,j} = 0.015$, "high" where $h_{min,j} = 0.016$, "low" where $h_{min,j} = 0.032$ and "very low" where $h_{min,j} = 0.040$.

As a next step, we estimate the standard error $\hat{\sigma}_m$ [25] of the estimated density $\hat{f}(y_m)$, which was computed with a Fast Fourier Transform.

Step 2. Determine grid points assigned to background.

We are no longer considering the original background test described in step 1 of section 2.3 in our original publication [25].

Our current approach involves two methods to background assignment. For cluster detail levels high, medium, and low, DBM assigns grid points to background if

$$b^2 * g < n * f^3 + (b^2 - 1) * f^2 \qquad (1);$$

Where $b=4.3$ for high and medium cluster detail level and 4 for low cluster detail level;

$$g(y_{\mathbf{m}}) = \frac{1}{n}\sum_{l_1=-Z_1}^{Z_1}\sum_{l_2=-Z_2}^{Z_2} w_{\mathbf{m-l}} \times \prod_{j=1}^{2}\left(\frac{\phi(l_j\Delta_j/h_j)}{h_j}\right)^2 \qquad (2);$$

f is the density computed as described in [25]; n is the count of events/cells in the 2D projection DBM is clustering.

For very high and very low cluster detail level, assigning background is done by ordering the M^2 grid points as y_i so that $\hat{f}(y_1) <= \hat{f}(y_2) <= \ldots <= \hat{f}(y_{M^2})$ and then assigning the grid points y_i less than a percent threshold to the background until a

certain proportion of cells have been assigned. For "very high", the threshold is 1.5 percent of the most dense grid point; and, for "very low", it is 4 percent.

Step 3. Construct association pointers. S is the set of grid points where the density is significantly different from zero. Here we create, for all grid points y_m; $m \in S$, in turn, a directional association with at least one other grid point using an association rule. From each grid point y_m, $m \notin S$, a pointer is established that points to the background state. Consider all the neighboring grid points $p_1, \ldots, p_{n_m}$, which are defined as the set of all grid points (excluding y_m) contained in the box $\bigcap_{j=1}^{2} \{x : y_{mj} - \Delta_j \le x_j \le y_{mj} + \Delta_j\}$. Define the corner neighbors to be the neighboring grid points x of y_m with $x_1 \ne y_{m1}$ and $x_2 \ne y_{m2}$. Define a function $\hat{s}$ on the neighboring grid points by

$$\hat{s}(p_i) = \hat{f}(p_i) \qquad (3)$$

if p_i is not a corner neighbor; and

$$\hat{s}(p_i) = \hat{f}(y_m) + \frac{1}{\sqrt{2}}(\hat{f}(p_i) - \hat{f}(y_m)) \qquad (4)$$

if p_i *is* a corner neighbor.

Let $p \in \{p_1, \ldots, p_{n_m}\}$ such that $\hat{s}(p) = max_{k=1,\ldots,n_m} \hat{s}(p_k)$, splitting ties arbitrarily. Then establish an association pointer from y_m to p provided the following two conditions hold: $\hat{f}(p) > \hat{f}(y_m)$ and $\frac{\partial}{\partial e}\hat{f}(y_m) > 0$, where $e = \frac{p - y_m}{\|p - y_m\|}$, $||\cdot||$ denotes Euclidean norm, and $\frac{\partial}{\partial e}\hat{f}(y_m)$ is defined as follows:

$$\frac{\partial}{\partial e}\hat{f}(y_m) = \sum_{a=1}^{2} e_a \frac{\partial}{\partial y_{m_a}} \hat{f}(y_m) \qquad (5)$$

$$\frac{\partial}{\partial y_{m_a}}\hat{f}(y_m)=\frac{1}{n}\sum_{l_1=-Z_1}^{Z_1}\sum_{l_2=-Z_2}^{Z_2} w_{m-l}\cdot\frac{-l_a\Delta_a}{h_a^2}\prod_{j=1}^{2}\frac{\varphi(\frac{l_j\Delta_j}{h_j})}{h_j}.$$

Here e_1, e_2 denotes the standard Euclidean basis vectors and $e = (e_1, e_2)$. Since not every cluster will have a unique local maximum, there is a merging process addressed in ***Step 5***.

Step 4. Assign grid points to cluster or background. Here we follow directional associations between grid points to determine terminal states for one or more pointer paths. Then we assign each data item to a grid point according to an assignment rule and use terminal states of said pointer paths to determine a cluster for said data item.

For all grid points y_m; $m \in S$, in turn: if a pointer originates at y_m, then it points to a different grid point, which itself might have a pointer originating from it. The succession of pointers is followed until one arrives at a grid point y_z such that either

(a) y_z has no pointer originating from it, or

(b) y_z has a pointer to the background state or to a cluster state.

In case (a), if there is no pointer into y_z (this implies that $y_m = y_z$), no pointers are removed or established. If however, case (a) holds and there is a pointer into y_z, then all the pointers visited in succession are removed and new pointers originating from each grid point visited in succession will be established to the background state, provided the following condition holds:

$$\hat{f}(y_z) < q(0.95^{\frac{1}{\kappa}})\sqrt{\hat{\sigma}_z^2} \tag{6}$$

$$\kappa = \frac{|S| \sum_{m \in S} w_m}{2\pi n \prod_{j=1}^{2} h_j \sum_{m \in S} \hat{f}(y_m)}$$

Here $|S|$ is the size of the set S and $q(x)$ denotes the (100x)-th percentile of the standard normal distribution. Otherwise, a new pointer is established that originates from y_z and points to a newly established cluster state.

In case (b), no pointers are removed or established.

Step 5. Merge clusters not separated by a statistically significant trough. Let $y_{m(1)}, \dots, y_{m(k)}$ be the set of all grid points which have a pointer originating from them to a cluster state, enumerated such that $\hat{f}(y_{m(1)}) \geq \dots \geq \hat{f}(y_{m(k)})$.

For i=1,...,k do the following:

Set $\mathrm{A} = \{m(i)\}$. Iterate the following loop until no more indices are added to A:

(Begin loop)

For each index $\boldsymbol{a} \in A$ in turn, add all the indices $\boldsymbol{p}$ to A that satisfy

(1) y_p is a neighbor of y_a as defined in ***Step 3***;

(2) no pointer originates from y_p,

(3) $\hat{f}(y_p) + \hat{\sigma}_p \geq \hat{f}(y_{m(i)}) - \hat{\sigma}_{m(i)}$.

(End loop)

Denote by B the set containing $m(i)$ as well as the indices of grid points which satisfy the following two conditions. The grid point possesses a pointer to a cluster state, and the grid point has some y_p , $p \in A$ as neighbor. Define q by, $\hat{f}(y_q) = max_{r \in B} \hat{f}(y_r)$ breaking ties arbitrarily.

Denote by C the set of indices of grid points which satisfy the following two conditions. The grid point possesses a pointer to another grid point, and the grid point is a neighbor of y_q . If C is non-empty, define k by $\hat{f}(y_k) = max_{r \in C} \hat{f}(y_r)$, breaking ties arbitrarily. If C is non-empty and $\hat{f}(y_k) > \hat{f}(y_q)$, establish a new pointer from each y_p, $p \in \mathrm{A} \setminus \{\mathrm{m(i)}\}$, to the target of the pointer originating at y_k. Furthermore, for each $r \in B$, remove the pointer from y_r to the cluster state and establish a new pointer from y_r to the target of the pointer originating at y_k. Otherwise, if C is empty or $\hat{f}(y_k) \leq \hat{f}(y_q)$, establish a new pointer from each y_p, $p \in \mathrm{A} \setminus \{\mathrm{m(i)}\}$, to the target of the pointer originating at y_q. Furthermore, for each $r \in \mathrm{B} \setminus \{\mathrm{q}\}$, remove the pointer from y_r to the cluster state and establish a new pointer from y_r to the target of the pointer originating at y_q.

Step 6. Clusters merging iteration. Repeat ***Step 5*** until there are no more additions or deletions of pointers to cluster states.

Step 7. Assign grid points to the background. From each grid point that does not have a pointer originating from it, establish a pointer pointing to the background state.

References

1. Saeys, Y., Gassen, S.V., Lambrecht, B.N. Computational flow cytometry: helping to make sense of high-dimensional immunology data. Nat Rev Immunol. 16(7), 449-62 (2016). doi: 10.1038/nri.2016.56.

2. Hastie, T., Tibshirani, R., Friedman, J. The elements of statistical learning. (Springer-Verlag, 2009).

3. Orlova, D. Y., Herzenberg, L. A., Walther, G. Science not art: statistically sound methods for identifying subsets in multi-dimensional flow and mass cytometry datasets. Nat Rev Immunol. 18 (1), 77 (2018). doi:10.1038/nri.2017.150.

4. Silverman, B. Density estimation for statistics and data analysis. (Chapman & Hall/CRC, 1986).

5. Newell, E.W., Cheng, Y. Mass cytometry: blessed with the curse of dimensionality. Nat Immunol. 17(8), 890-5 (2016). doi: 10.1038/ni.3485.

6. Scott, D.W. Multivariate Density Estimation - Theory, Practice and Visualization. (Wiley, 1992).

7. Implementation of Density-Based Spatial Clustering of Applications with Noise (DBSCAN) in MATLAB. Version 1.0. September 2015. Available at https://www.mathworks.com/matlabcentral/fileexchange/52905-dbscan-clustering-algorithm . Date accessed: June 2017.

8. Aghaeepour, N., Nikolic, R., Hoos, H.H., Brinkman, R.R. Rapid cell population identification in flow cytometry data. Cytometry A. 79(1), 6-13 (2011). doi: 10.1002/cyto.a.21007.

9. GenePattern. Freely available computational biology open-source software package developed at the Broad Institute of MIT and Harvard. Available at http://software.broadinstitute.org/cancer/software/genepattern/flow-cytometry-gating-and-clustering . Date accessed: June 2017.

10. Samusik, N., Good, Z., Spitzer, M.H., Davis, K.L., Nolan, G.P. Automated mapping of phenotype space with single-cell data. Nature Methods 13(6), 493–6 (2016). doi: 10.1038/nmeth.3863.

11. Samusik, N. VorteX Clustering Environment. Available at http://web.stanford.edu/~samusik/vortex/ . Latest Release: Vortex 21-Apr-2017. Date accessed: June 2017.

12. Bellman, R.E. Adaptive control processes: a guided tour. (Princeton Press, 1961).

13. Qian, Y., Wei, C., Eun-Hyung Lee, F., Campbell, J., Halliley, J., Lee, J.A., Cai, J., Kong, Y.M., Sadat, E., Thomson, E., Dunn, P., Seegmiller, A.C., Karandikar, N.J., Tipton, C.M., Mosmann, T., Sanz, I., Scheuermann, R.H. Elucidation of seventeen human peripheral blood B-cell subsets and quantification of the tetanus response using a density-based method for the automated identification of cell populations in multidimensional flow cytometry data. Cytometry B Clin. Cytom. 78 Suppl 1, S69–82 (2010). doi: 10.1002/cyto.b.20554.

14. Anchang, B., Hart, T.D., Bendall, S.C., Qiu, P., Bjornson, Z., Linderman, M., Nolan, G.P., Plevritis, S.K. Visualization and cellular hierarchy inference of single-cell data using SPADE. Nat Protoc. 11(7), 1264-79 (2016). doi: 10.1038/nprot.2016.066.

15. Lo, K., Hahne, F., Brinkman, R.R., Gottardo, R. flowClust: a Bioconductor package for automated gating of flow cytometry data. BMC Bioinformatics. 10: 145 (2009). doi: 10.1186/1471-2105-10-145.

16. Pyne, S., Hu, X., Wang, K., Rossin, E., Lin, T.I., Maier, L.M., Baecher-Allan, C., McLachlan, G.J., Tamayo, P., Hafler, D.A., De Jager, P.L., Mesirov, J.P. Automated high-dimensional flow cytometric data analysis. Proc. Natl Acad Sci USA. 106(21), 8519–24 (2009). doi: 10.1073/pnas.0903028106.

17. Naim, I., Datta, S., Sharma, G., Cavenaugh, J.S., Mosmann, T.R. "SWIFT: Scalable weighted iterative sampling for flow cytometry clustering"; IEEE International Conference on Acoustics Speech and Signal Processing (ICASSP). 509-12 (2010).

18. van der Maaten, L., Hinton, G. Visualizing data using t-SNE. Journal of Machine Learning Research. 9, 2579–2605 (2008).

19. Wattenberg, M., Viegas, F., Johnson, I. How to Use t-SNE Effectively. Distill, 2016. Available at http://distill.pub/2016/misread-tsne/ . Date accessed: June 2017.

20. Levina, E., Bickel, P. Maximum likelihood estimation of intrinsic dimension. In Advances in NIPS. 17. MIT Press, 2005.

21. Ozakin, A., Gray, A. Submanifold density estimation. Advances in neural information processing systems, 2009.

22. Lombardi, G. Intrinsic dimensionality estimation techniques. Version 1.1. May 2013. Available at https://www.mathworks.com/matlabcentral/fileexchange/40112-intrinsic-dimensionality-estimation-techniques?focused=3785388&tab=example . Date accessed: June 2017.

23. Chen, H., Lau, M.C., Wong, M.T., Newell, E.W., Poidinger, M., Chen, J. Cytofkit: A Bioconductor Package for an Integrated Mass Cytometry Data Analysis Pipeline. PLoS Comput. Biol. 12:e1005112 (2016).

24. GenePattern. Freely available computational biology open-source software package developed at the Broad Institute of MIT and Harvard. Available at http://software.broadinstitute.org/cancer/software/genepattern/flow-cytometry-gating-and-clustering . Date accessed: November 2017.

25. Walther, G., Zimmerman, N., Moore, W., Parks, D., Meehan, S., Belitskaya, I., Pan, J., Herzenberg, L. Automatic clustering of flow cytometry data with density-based merging. Adv. Bioinformatics. 686759 (2009). doi: 10.1155/2009/686759.

26. Rahmah, N., Sitanggang, I.S. Determination of optimal Epsilon (Eps) value on DBSCAN algorithm to clustering data on peatland hotspots in Sumatra. IOP Conference Series: Earth and Environmental Science 31 (2016); doi:10.1088/1755-1315/31/1/012012

27. Choosing parameters of DBSCAN algorithm. June 2014. Available at https://github.com/alitouka/spark_dbscan/wiki/Choosing-parameters-of-DBSCAN-algorithm . Date accessed: June 2017.

28. Ghosn, E.E.B., Cassado, A.A., Govoni, G.R., Fukuhara, T., Yang, Y., Monack, D.M., Bortoluci, K.R., Almeida, S.R., Herzenberg, L.A., Herzenberg, L.A. Two physically, functionally, and developmentally distinct peritoneal macrophage subsets. Proc. Natl. Acad. Sci. U. S. A. 107(6), 2568–73 (2009). doi: 10.1073/pnas.0915000107.
