# Supplementary material for: Automated subset identification and characterization pipeline for multidimensional flow and mass cytometry data clustering and visualization
Source: Commun Biol. 2019 Jun 20;2:229. doi: 10.1038/s42003-019-0467-6 (PMC6586874; doi:10.1038/s42003-019-0467-6)
Supplement: Supplementary file 2 — Description of Additional Supplementary Files [file 42003_2019_467_MOESM2_ESM.docx]

Supplementary data files contain synthetic data sets (in text format) underlying Figures 2-4.

supplementary data 1 – data for Sample A.

supplementary data 2 – data for Sample B.
