# Supplementary material for: Automated subset identification and characterization pipeline for multidimensional flow and mass cytometry data clustering and visualization
Source: Commun Biol. 2019 Jun 20;2:229. doi: 10.1038/s42003-019-0467-6 (PMC6586874; doi:10.1038/s42003-019-0467-6)
Supplement: Supplementary file 5 — Reporting Summary [file 42003_2019_467_MOESM5_ESM.pdf]

nature research

Corresponding author(s): Darya Orlova
Last updated by author(s): May 13, 2019

Reporting Summary

Nature Research wishes to improve the reproducibility of the work that we publish. This form provides structure for consistency and transparency in reporting. For further information on Nature Research policies, see Authors & Referees and the Editorial Policy Checklist.

Statistics

For all statistical analyses, confirm that the following items are present in the figure legend, table legend, main text, or Methods section.

n/a	Confirmed	
☐	☒	The exact sample size (*n*) for each experimental group/condition, given as a discrete number and unit of measurement
☐	☒	A statement on whether measurements were taken from distinct samples or whether the same sample was measured repeatedly
☒	☐	The statistical test(s) used AND whether they are one- or two-sided<br>*Only common tests should be described solely by name; describe more complex techniques in the Methods section.*
☒	☐	A description of all covariates tested
☒	☐	A description of any assumptions or corrections, such as tests of normality and adjustment for multiple comparisons
☐	☒	A full description of the statistical parameters including central tendency (e.g. means) or other basic estimates (e.g. regression coefficient) AND variation (e.g. standard deviation) or associated estimates of uncertainty (e.g. confidence intervals)
☒	☐	For null hypothesis testing, the test statistic (e.g. *F*, *t*, *r*) with confidence intervals, effect sizes, degrees of freedom and *P* value noted<br>*Give P values as exact values whenever suitable.*
☒	☐	For Bayesian analysis, information on the choice of priors and Markov chain Monte Carlo settings
☒	☐	For hierarchical and complex designs, identification of the appropriate level for tests and full reporting of outcomes
☒	☐	Estimates of effect sizes (e.g. Cohen's *d*, Pearson's *r*), indicating how they were calculated

Our web collection on statistics for biologists contains articles on many of the points above.

Software and code

Policy information about availability of computer code

Data collection	DiVa (http://www.bdbiosciences.com)
Data analysis	The analysis pipeline described in our manuscript is implemented in AutoGate (www.cytonegie.org) software package that supports graphical user interface; Python source code is available at https://github.com/dyorlova/QFMatch_MDS_dendrogram .<br><br>To test the performance of other methods we used:<br>DBSCAN in MATLAB (https://www.mathworks.com/matlabcentral/fileexchange/52905-dbscan-clusteringalgorithm). Date accessed: June 2017.<br>VorteX 21 (http://web.stanford.edu/~samusik/vortex/). Date accessed: June 2017.<br>Rphenograph, ClusterX, DensVM (https://bioconductor.org/packages/cytofkit/). Date accessed: November 2017.<br>flowMeans (http://software.broadinstitute.org/cancer/software/genepattern/flow-cytometry-gatingand-clustering). Date accessed: June 2017.<br>t-SNE plugin provided by FlowJo v10 (https://www.flowjo.com/). Date accessed: July 2018.<br>SPADE v3.0 (http://pengqiu.gatech.edu/software/SPADE/) Date accessed: July 2018.

For manuscripts utilizing custom algorithms or software that are central to the research but not yet described in published literature, software must be made available to editors/reviewers. We strongly encourage code deposition in a community repository (e.g. GitHub). See the Nature Research guidelines for submitting code & software for further information.

October 2018

Data

Policy information about availability of data

All manuscripts must include a data availability statement. This statement should provide the following information, where applicable:

- Accession codes, unique identifiers, or web links for publicly available datasets
- A list of figures that have associated raw data
- A description of any restrictions on data availability

The datasets generated during and/or analyzed during the current study (Figures 5-8) are available in the FlowRepository and Cytobank:

https://flowrepository.org/id/RvFrin9QJBrBl7euVYafJg8MBtow5TSn0Cbf6ibJFTQbutUCP8VbTKi70DJD7TJg

https://flowrepository.org/id/RvFrmp0uY05bFrRfQW6XgcLV360pTCjz5ieEKzaHHGsTDoWEWpBspy21QVrQhFxz

https://flowrepository.org/id/RvFr85dLvWpwdnNDGBkj5qCB8skivxce0qGYsDNtsb52uflvb6C21xDjujsOXnY8

https://www.cytobank.org/nolanlab/reports/Levine2015.html

Field-specific reporting

Please select the one below that is the best fit for your research. If you are not sure, read the appropriate sections before making your selection.

☒ Life sciences ☐ Behavioural & social sciences ☐ Ecological, evolutionary & environmental sciences

For a reference copy of the document with all sections, see nature.com/documents/nr-reporting-summary-flat.pdf

Life sciences study design

All studies must disclose on these points even when the disclosure is negative.

Sample size	Data were collected for about 0,5-1,0x10^6 cells to sufficiently represent major cell subsets.
Data exclusions	No data were excluded.
Replication	All attempts at replication were successful.
Randomization	Random healthy controls were chosen.
Blinding	Blinding was not relevant since only one group of samples was used.

Reporting for specific materials, systems and methods

We require information from authors about some types of materials, experimental systems and methods used in many studies. Here, indicate whether each material, system or method listed is relevant to your study. If you are not sure if a list item applies to your research, read the appropriate section before selecting a response.

Materials & experimental systems

n/a	Involved in the study	
☐	☒	Antibodies
☒	☐	Eukaryotic cell lines
☒	☐	Palaeontology
☒	☐	Animals and other organisms
☐	☒	Human research participants
☒	☐	Clinical data

Methods

n/a	Involved in the study	
☒	☐	ChIP-seq
☐	☒	Flow cytometry
☒	☐	MRI-based neuroimaging

Antibodies

Antibodies used

Marker	Clone	Fluorochrome	Supplier	Catalog #
B220	R3A-6B2	PE	BD Biosciences	553090
CD5	UCHT2	PE-Cy5	BD Biosciences	555354
CD10	HI10a	PerCP-Cy5.5	BD Biosciences	563508
CD19	HIB19	BV785	BioLegend	302240
CD20	2H7	BV650	BioLegend	302336
CD23	EBVCS-5	APC-Cy7	BioLegend	338520

CD27	M-T271	BV421	BioLegend	356418
CD38	HIT2	APC	BD Biosciences	555462
CD43	L60	Alexa Fluor 700 (in-house conjugation)	BD Biosciences	551457
CD95	DX2	BV605	BioLegend	305628
CD132	AG184	BV711	BD Biosciences	563129
CD3	UCHT1	BV570	BioLegend	300436
CD14	M5E2	BV570	BioLegend	301832
CD16	3G8	BV570	BioLegend	302036
CD45	HI30	Alexa Fluor 488	BioLegend	304017
IgD	IA6-2	PE-Cy7	BD Biosciences	561314
IgM	G20-127	PE-CF594	BD Biosciences	562539
Zombie Aqua	n/a	Aqua	BioLegend	423102

Marker	Clone	Fluorochrome	Supplier	Catalog #
CD117(c-Kit)	YB5.B8	PE-CF594	BD Biosciences	562407
CD3	UCHT1	Alexa Fluor 647	BioLegend	300416
CD14	HCD14	Alexa Fluor 647	BioLegend	325612
CD16	3G8	Alexa Fluor 647	BioLegend	302020
CD5	UCHT2	Brilliant Violet 711	BD Biosciences	563170
CD19	HIB19	Brilliant Violet 785	BioLegend	302240
CD34	8G12	PE	BD Biosciences	345802
CD38	HIT2	PerCP-Cy5.5	BD Biosciences	551400
CD41a	HIP8	PE-Cy5	BD Biosciences	559768
CD43	1G10	APC-H7	BD Biosciences	655407
CD45	HI30	Brilliant Violet 650	BioLegend	304044
CD49f	GoH3	Brilliant Violet 421	BD Biosciences	747725
CD90(Thy-1)	5E10	PE-Cy7	BioLegend	328124
CD133	AC133	VioBright FITC	Miltenyi Biotec	130-113-673
CD135(Flt-3/Flk-2)	BV10A4H2	Biotin (Qdot 605-SA)	BioLegend	313312
IgD	IA6-2	Alexa Fluor 700	BioLegend	348230
IgM	MHM-88	Brilliant Violet 570	BioLegend	314517
Zombie Aqua Viability dye		Aqua Amine	BioLegend	423102
Qdot 605 Streptavidin	N/A	Qdot 605	ThermoFisher	Q10101MP

Validation

The optimal antibody concentration was determined by in-house serial titration using human total peripheral blood.

Human research participants

Policy information about studies involving human research participants

Population characteristics

Healthy volunteers

Recruitment

Blood was collected from healthy adults after informed consent. Fresh bone marrow mononuclear cells from adult healthy donors were obtained commercially from AllCells, LLC (Quincy, MA, Cat # ABM024).

Ethics oversight

Institutional Review Board

Note that full information on the approval of the study protocol must also be provided in the manuscript.

Flow Cytometry

Plots

Confirm that:

☐ The axis labels state the marker and fluorochrome used (e.g. CD4-FITC).

☒ The axis scales are clearly visible. Include numbers along axes only for bottom left plot of group (a 'group' is an analysis of identical markers).

☒ All plots are contour plots with outliers or pseudocolor plots.

☒ A numerical value for number of cells or percentage (with statistics) is provided.

Methodology

Sample preparation

The human peripheral blood dataset (Figure 6) was generated using a combination of 16 monoclonal antibodies (Hi-D 18-parameter flow cytometry panel): B220-PE, CD5-PECy5, CD10-PECy5.5, CD19-BV786, CD20-BV650, CD23-APCCy7, CD27-BV421, CD38-APC, CD43-AF700, CD95-BV605, CD132-BV711, CD3/CD14/CD16 (Dump)-BV570, CD45-AF488, IgD-PECy7, IgM-PECF594, and Aqua Amine (viability). After informed consent, 10 mL of peripheral blood was drawn in evacuated tubes containing EDTA (K2) (Vacutainer, BD Biosciences). The blood samples from healthy adult volunteers were collected, de-identified, and kindly provided by the Clinical and Translational Discovery Core (Biorepository) at Emory University and Children's Healthcare of Atlanta (http://www.pedsresearch.org/research/cores/biorepository). These studies were exempt from the IRB review because they do not meet the definition of clinical investigation or research with human subjects. Data was collected for about 0,5x10^6 cells.

The human bone marrow dataset (Figure 7) was generated using a combination of 16 monoclonal antibodies (Hi-D 18-parameter flow cytometry panel): CD133-VioBright, CD49f-BV421, IgM-BV570, CD135-biotin (Qdot 605-SA), CD45-BV650, CD5-BV711, CD19-BV786, CD3/CD14/CD16 (Dump)-AF647, IgD-AF700, CD43-APC-Cy7, CD34-PE, C-Kit-PECF594, CD41-PE-Cy5, CD38-PE-Cy5.5, CD90-PE-Cy7, Zombie Aqua. Fresh bone marrow mononuclear cells from adult healthy donors were obtained commercially from AllCells, LLC (Quincy, MA, Cat # ABM024). Data was collected for about 0,5-1,0x10^6 cells.

Instrument	Human peripheral blood and bone marrow cells were analyzed on the BD LSRII instrument (5-laser, 18-color) at the Emory's Pediatrics/Winship Flow Cytometry Core.
Software	The analysis pipeline described in our manuscript is implemented in AutoGate (www.cytonegie.org) software package that supports graphical user interface; Python source code is available at https://github.com/dyorlova/QFMatch_MDS_dendrogram
Cell population abundance	Cell populations abundance is presented on the relevant figures. No sorting procedure was involved in this study.
Gating strategy	See figures for gating sequences.

☒ Tick this box to confirm that a figure exemplifying the gating strategy is provided in the Supplementary Information.
